# Real-world outcomes with elranatamab in multiple myeloma: a multicenter analysis from the U.S. Multiple Myeloma Immunotherapy Consortium

**DOI:** 10.1038/s41408-026-01477-z

**Published:** 2026-03-27

**Authors:** Andrew J. Portuguese, James A. Davis, Shahzad Raza, Omar Castaneda Puglianini, Ciara Freeman, Melissa Alsina, Corbin Wright, Brandon J. Blue, Rachid C. Baz, Kenneth H. Shain, Utkarsh Goel, Jack Khouri, Faiz Anwer, Hossam M. Ali, Oren Pasvolsky, Mahmoud Gaballa, Krina Patel, Hector Garcia-Pleitez, Damian Mikulski, Lindsay Fogel, Eli Zolotov, Aishwarya Sannareddy, Aimaz Afrough, Larry D. Anderson, Kelley Julian, Danai Dima, Rahul Banerjee, Emily C. Liang, Raffaella Cassano Cassano, Megan M. Herr, Hamza Hassan, Leyla Shune, Jeries Kort, Shonali Midha, Omar Nadeem, Surbhi Sidana, Yi Lin, Frederick L. Locke, Doris K. Hansen, Douglas Sborov, Noa Biran, Ariel Grajales-Cruz

**Affiliations:** 1 Fred Hutchinson Cancer Center, Seattle, WA USA; 2 Medical University of South Carolina, Charleston, SC USA; 3 Cleveland Clinic, Cleveland, OH USA; 4 H. Lee Moffitt Cancer Center and Research Institute, Tampa, FL USA; 5 MD Anderson Cancer Center, Houston, TX USA; 6 Medical University of Lodz, Lodz, Poland; 7 Hackensack Meridian Health, Hackensack, NJ USA; 8 UT Southwestern Medical Center, Dallas, TX USA; 9 Huntsman Cancer Institute, University of Utah, Salt Lake City, UT USA; 10 Roswell Park Comprehensive Cancer Center, Buffalo, NY USA; 11 University of Kansas Medical Center, Kansas City, KS USA; 12 Dana-Farber Cancer Institute, Boston, MA USA; 13 Stanford University School of Medicine, Stanford, CA USA; 14 Mayo Clinic, Rochester, MN USA

**Keywords:** Myeloma, Immunotherapy

## Abstract

Elranatamab, a BCMA-CD3 bispecific antibody, has demonstrated robust activity in relapsed/refractory multiple myeloma (RRMM), but real-world outcomes remain poorly defined. We conducted a multicenter retrospective study of 130 patients treated with commercial elranatamab across nine U.S. academic centers. The cohort was heavily pretreated (91% triple-class refractory, 49% penta-refractory), with 49% previously exposed to BCMA-targeted therapies. Only 22% would have met eligibility for MagnetisMM-3 cohort A. The overall response rate (ORR) was 65%, including ≥CR in 36%. Median progression-free survival (PFS) and overall survival (OS) were 4.3 and 14.6 months, respectively, shorter than MagnetisMM-3. Elevated LDH and low hemoglobin independently predicted poor outcomes and were incorporated into the novel ALPS (Anemia-LDH Prognostic System) score, which stratified patients into distinct risk groups for ORR, OS, PFS, and duration of response. Prior BCMA exposure reduced depth of response, with inferior OS observed in those treated within one year of prior therapy. Infections occurred in 38% of patients. Intravenous immunoglobulin supplementation, modeled as a time-dependent covariate, was associated with improved infection-free survival and PFS. While the incidence of CRS was modestly lower than in MagnetisMM-3, ICANS occurred more frequently in this real-world cohort. These findings highlight the efficacy, limitations, and supportive care needs of elranatamab in a frailer, more heterogeneous real-world RRMM population.

## Introduction

B-cell maturation antigen (BCMA)-targeted bispecific antibodies (BsAbs) have emerged as a transformative therapeutic class for relapsed or refractory multiple myeloma (RRMM), offering deep responses in heavily pretreated patients. Elranatamab, a humanized BCMA×CD3 bsAb, demonstrated encouraging activity in this setting. In the registrational Phase 2 MagnetisMM-3 trial, elranatamab monotherapy demonstrated an overall response rate (ORR) of 61% and a complete response (CR) rate of 35% in patients who were BCMA-naïve and triple-class-refractory [1]. Long-term follow-up confirmed the durability of these responses, with median progression-free survival (PFS) of 17.2 months, overall survival (OS) of 24.6 months, and no new safety signals despite transition to less frequent dosing [2, 3].

However, clinical trial populations often underrepresent frail patients or those with prior BCMA-directed therapy. Real-world experience with elranatamab remains limited, particularly with regard to treatment durability, toxicity, and supportive care interventions such as intravenous immunoglobulin (IVIg) supplementation. Infections are a major cause of morbidity and mortality in patients treated with BCMA-directed BsAbs, driven by profound hypogammaglobulinemia, cumulative immunosuppression, and treatment-induced cytopenias. Preliminary retrospective studies suggest that IVIg use may reduce serious infections and improve infection-free survival (IFS), but data are sparse and heterogeneous [4, 5].

In this multicenter retrospective analysis from the U.S. Multiple Myeloma Immunotherapy Consortium, we report real-world outcomes of 130 patients treated with commercial elranatamab across nine academic institutions. We characterize the depth and durability of response, and assess key endpoints including PFS, OS, duration of response (DOR), and IFS. We also evaluate clinical and laboratory factors associated with outcomes and explore the impact of IVIg prophylaxis using time-dependent covariate modeling.

## Subjects and methods

### Patients

We conducted a multicenter, retrospective cohort study of patients treated with commercial elranatamab from 8/2023–3/2025 at nine academic centers (Moffitt Cancer Center, Cleveland Clinic, MD Anderson Cancer Center, Hackensack Meridian Health, UT Southwestern Medical Center, Huntsman Cancer Institute, Fred Hutchinson Cancer Center, Roswell Park Comprehensive Cancer Center, and University of Kansas Medical Center).

### Ethics approval and consent to participate

This multicenter retrospective study involving human participants was conducted in accordance with the relevant guidelines and regulations, including the Declaration of Helsinki. Approval was obtained from the Institutional Review Board at each participating center. Informed consent was obtained in accordance with local institutional requirements. No identifiable images of human research participants are included in this manuscript.

### Definitions

Treatment response was assessed per the International Myeloma Working Group uniform response criteria [6]. The number of prior lines of therapy (LOTs) was determined according to published guidelines [7]. High-risk cytogenetic abnormalities were defined as gain/amp(1q), del(17p), t(4;14), t(14;16). Cytokine release syndrome (CRS) and immune effector cell-associated neurotoxicity (ICANS) were graded per American Society for Transplantation and Cellular Therapy (ASTCT) criteria [8]. CAR-HEMATOTOX scores were calculated as previously described [9].

### Statistical analysis

All statistical analyses were conducted using R Statistical Software (Version 4.4.3; R Core Team 2025) [10]. Continuous variables were analyzed using the Wilcoxon rank-sum test, while proportions were compared using Pearson’s Chi-squared test or Fisher’s exact test. PFS events were defined as death, relapse, or progressive disease (PD). DOR was calculated as the time to relapse, progression, or death for patients who had at least a partial response (PR) after initial response to therapy. Kaplan-Meier (KM) estimates were employed to assess OS, PFS, and DOR. Median follow-up time was estimated with the reverse KM method.

Duration of therapy was defined as the time from the first elranatamab dose to permanent discontinuation or death. For patients who remained on treatment at the time of data cutoff, duration was treated as right-censored at the date of last elranatamab administration. This approach, implemented using Kaplan-Meier methods, avoids underestimating treatment duration by accounting for patients with ongoing therapy.

Logistic and Cox regression were used for binary and time-to-event outcomes, respectively. Multivariable analysis (MVA) adjusted for prior LOTs, baseline Hgb (per g/dL), and LDH (per 100 U/L) as continuous variables; and for BCMA exposure, extramedullary disease (EMD; extraosseous soft tissue lesions), and Eastern Cooperative Oncology Group performance status (ECOG PS) ≥ 2 as binary variables. Baseline ferritin (per 1000 ng/mL) was evaluated in univariate, but not MVA, due to missingness (data not available for 19 patients).

Because patients initiated IVIg at varying times, this was modeled as a time-dependent covariate (TVC). Associations between IVIg utilization and time-to-event outcomes were evaluated using time-dependent Cox models. For visualization, a novel survival trajectories plot (Smith-Zee plot) based on the time-dependent Cox model was generated. These curves represent the survival of a hypothetical patient with a pre-specified time of TVC status change [11, 12]. Kaplan-Meier curves were not used, as they assume treatment assignment (IVIg vs. No IVIg) is fixed at baseline, which introduces immortal time bias.

We evaluated non-linear relationships between continuous variables and clinical outcomes as previously described [13]. The continuous variable was modeled as a restricted cubic spline with three knots to allow for flexible functional forms.

To assess the prognostic performance of each risk score, we fit Cox proportional hazards models for OS and PFS. Time-dependent area under the curve (AUC) and concordance indices (C-indices) were estimated at 1, 3, and 6 months using the Score() and cindex() functions from the riskRegression (v2025.9.17) and pec (v2025.6.24) packages, respectively. Predicted survival probabilities were obtained via predictRisk() and observed event probabilities were calculated within deciles of predicted risk using KM estimators. Calibration curves were generated by plotting mean predicted versus observed risk across deciles, with (locally estimated scatterplot smoothing) LOESS applied to visualize model calibration over time.

## Results

### Baseline characteristics

As shown in Table 1, the cohort included 130 patients with a median age of 71 yrs (range, 39–95), of whom 57% were female, 82% identified as White, 13% as Black. A substantial proportion (35%) had an ECOG PS of ≥2 including 11% with ECOG PS ≥ 3.

**Table 1:** Baseline characteristics of patients receiving elranatamab.

Characteristic	*N* = 130^a^
**Age (years)**	71 (39 to 95)
**Female sex**	74 (57%)
**Race**	
White	102 (82%)
Black	16 (13%)
AAPI	3 (2.4%)
American Indian	2 (1.6%)
Other	2 (1.6%)
**ECOG**	
0	22 (17%)
1	60 (48%)
2	30 (24%)
3+	14 (11%)
**Heavy chain**	
IgG	75 (58%)
IgA	28 (22%)
IgM	2 (1.5%)
IgD	2 (1.5%)
None	23 (18%)
**True EMD**	28 (22%)
**≥50% BMPCs**	15 (18%)
**Oligo or non-secretory**	12 (9.4%)
**≥1 HRCA**	85 (69%)
**High-risk cytogenetic abnormality at any time**	
**del(17p)**	31 (25%)
**t(4;14)**	17 (13%)
**t(14;16)**	8 (6.3%)
**gain/amp(1q)**	70 (56%)
**amp(1q)**	9 (7.9%)
**Prior LOTs**	6.00 (4.00, 8.00)
**Prior ASCT**	66 (51%)
**Prior CAR-T**	54 (42%)
**Refractory status**	
**Bortezomib refractory**	87 (67%)
**Lenalidomide refractory**	110 (85%)
**Pomalidomide refractory**	96 (74%)
**Anti-CD38 refractory**	116 (89%)
**Triple refractory**	118 (91%)
**Penta refractory**	64 (49%)
**Prior BCMA-directed therapy**	64 (49%)
**Most recent BCMA**	
CAR-T	54 (84%)
TCE	6 (9.4%)
ADC	4 (6.3%)
**Trial eligible**	28 (22%)
**Received full dose**	121 (93%)

^*a*^Median (Min to Max); *n* (%); Median (Q1, Q3).

*EMD* extramedullary disease, *BMPCs* bone marrow plasma cells, *HRCA* high-risk cytogenetic abnormality, *LOTs* lines of therapy, *ASCT* autologous stem cell transplant, *CAR-T* chimeric antigen receptor T-cell therapy, *TCE* T-cell engager, *ADC* antibody drug conjugate, *BCMA* B-cell maturation antigen.

Patients had received a median of six prior LOTs; 91% were triple-class refractory and 49% were penta-refractory. Nearly half (49%) had prior exposure to BCMA-directed therapies, most commonly anti-BCMA CAR-T cells (84%), followed by T-cell engagers (9.4%) and antibody-drug conjugates (6.3%). The median interval since last BCMA exposure was 15.5 mo (interquartile range [IQR] 11.8–20.5). Notably, only 22% would have met eligibility for MagnetisMM-3 cohort A, which excluded patients with prior BCMA therapy [1]. Common reasons for trial ineligibility included prior BCMA exposure (*n* = 21), hemoglobin <8 g/dL (*n* = 14), ECOG PS ≥ 3 (*n* = 22), creatinine clearance <30 mL/min (*n* = 8), platelets <25 K/μL (*n* = 7), and central nervous system involvement (*n* = 4).

High-risk cytogenetic abnormalities (HRCAs)—defined as gain/amp[1q], del[17p], *t*(4;14), or *t*(14;16)—were present in 69% of patients, with specific frequencies of 56%, 25%, 13%, 6.3%, respectively. Supplemental Fig. 1 depicts combinations and frequencies of HRCAs among all patients, responders, and non-responders. EMD was observed in 22% of the cohort. Bone marrow plasma cell (BMPC) burden at treatment initiation was available for 85 patients, of whom 18% (15/85) had a high burden (≥50%).

### Response and survival outcomes

After a median follow-up of 7.5 months, the estimated median duration of elranatamab treatment was 4.0 months (95% CI, 2.93–6.3) across the full cohort and 7.9 months (95% CI, 5.83–NA) among those achieving at least a very good partial response (≥VGPR). Treatment discontinuation due to toxicity or non-relapse mortality occurred in 12%, but was significantly less common among those receiving IVIg (5% vs. 17%, p = 0.031). Overall, 46% of patients ultimately received IVIg.

**Fig. 1:**
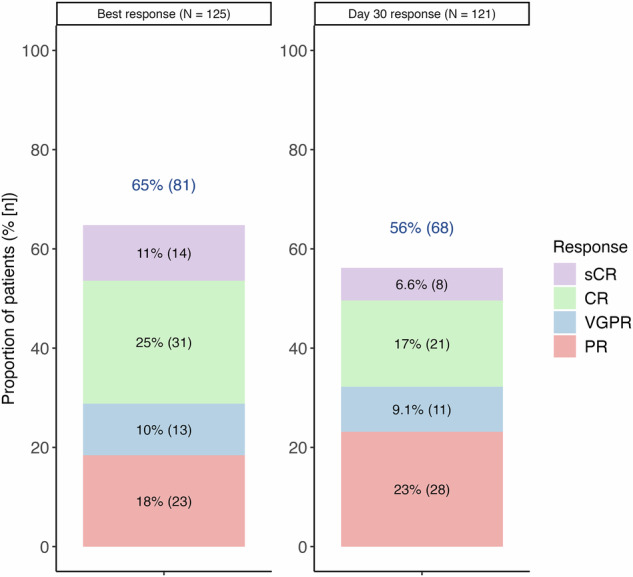
Best and day 30 response among evaluable patients.

**Fig. 2:**
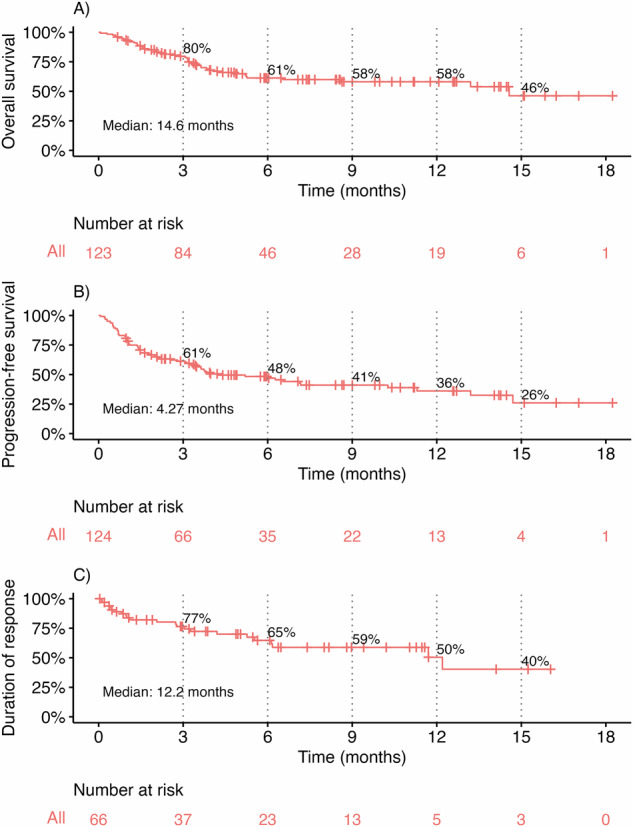
Survival outcomes in the overall cohort. Time to event outcomes among all patients, including **A** overall survival, **B** progression-free survival, and **C** duration of response.

As shown in Fig. 1, the overall response rate (ORR) was 65%, including ≥VGPR in 46% and ≥CR in 36%. Key time-to-event outcomes were as follows (Fig. 2): median PFS was 4.3 months (95% CI, 3.4–11.3), median OS was 14.6 months (95% CI, 8.6–NA), and median DOR was 12.2 months (95% CI, 6.1–NA).

**Fig. 3:**
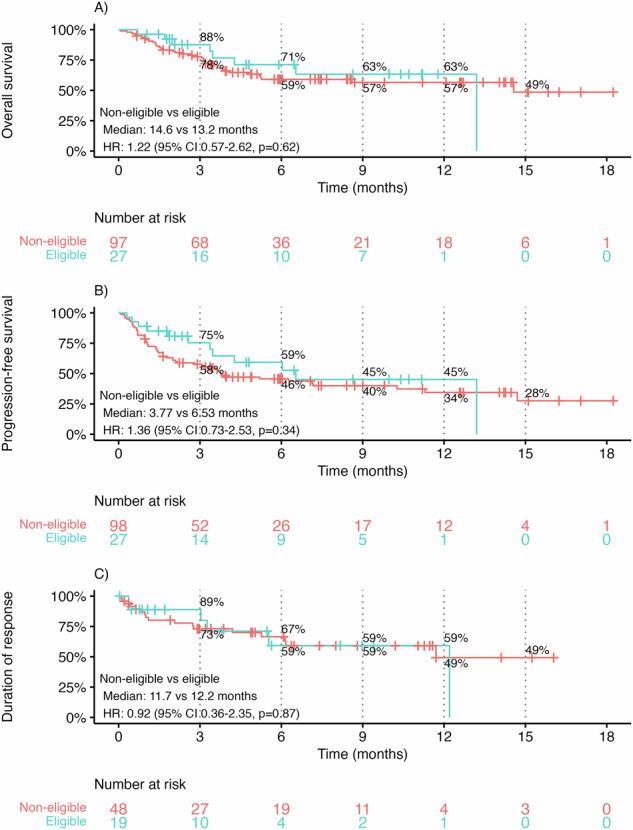
Survival outcomes by trial eligibility. Time to event outcomes stratified by trial eligibility, including **A** overall survival, **B** progression-free survival, and **C** duration of response.

Only a minority of patients (22%) would have met eligibility criteria for MagnetisMM-3 cohort A. As shown in Fig. 3, trial ineligibility had little impact on survival outcomes compared with eligible patients, with similar OS (median 14.6 vs. 13.2 months; HR 1.22, 95% CI 0.57–2.62, *p* = 0.62), PFS (median 3.77 vs. 6.53 months; HR 1.36, 95% CI 0.73–2.53, *p* = 0.34), and DOR (11.7 vs. 12.2 months; HR 0.92, 95% CI 0.36–2.35, *p* = 0.87).

### Predictors of response and survival

Full regression outputs from univariate and multivariable models—including predictors of response, toxicity, infection, and survival endpoints—are detailed in Supplementary Table 1.

Performance status significantly influenced outcomes. As shown in Supplementary Fig. 2, increasing ECOG PS (0-1 vs. 2 vs. 3+) was associated with shorter OS (median NA vs. NA. vs. 2.27 months, *p* < 0.001; 1-year: 89% vs. 68% vs. 42%) and PFS (median 6.5 vs. 2.1 vs. 1.1 months, p = 0.002; 1-year: 70% vs. 49% vs. 29%). Compared to ECOG PS 0–1, ECOG PS ≥ 2 was linked to reduced ORR (45% vs. 71%; OR 0.34, 95% CI 0.16–0.73, *p* = 0.006), shorter OS (median 4.3 months vs. NA; HR 2.41, 95% CI 1.33–4.35, *p* = 0.004), and shorter PFS (median 1.7 vs. 6.5 months; HR 1.77, 95% CI 1.09–2.87, *p* = 0.020).

Among patients with available baseline BMPC data (*n* = 85), a high marrow burden (≥50%) was associated with inferior outcomes. Compared with patients with <50% BMPCs, those with ≥50% BMPCs had lower response rates, including reduced ORR (40% vs. 73%; OR 0.25, 95% CI 0.07–0.78, *p* = 0.019) and ≥VGPR (20% vs. 57%; OR 0.19, 95% CI 0.04–0.65, *p* = 0.015). High BMPC burden was also associated with inferior survival, with shorter OS (median 3.2 months vs. not reached; HR 2.60, 95% CI 1.15–5.88, *p* = 0.022) and PFS (median 1.0 vs. 7.2 months; HR 3.39, 95% CI 1.73–6.66, *p* < 0.001). Consistent with a greater baseline disease burden, patients with ≥50% BMPCs also had lower baseline hemoglobin (median 8.8 vs. 10.2 g/dL, *p* = 0.020) and higher LDH levels (median 308 vs. 209 U/L, p = 0.009).

Prior exposure to BCMA-directed therapy did not significantly impact OS, PFS, or DOR (Supplementary Fig. 3). However, it was associated with lower odds of achieving ≥VGPR (OR 0.38, 95% CI 0.19–0.77, *p* = 0.008) and ≥CR (OR 0.43, 95% CI 0.20–0.89, *p* = 0.025). The depth of response to prior BCMA therapy was not correlated with elranatamab outcomes (Supplementary Fig. 4).

Among patients with available data on the interval between their most recent BCMA-directed treatment and initiation of elranatamab (*n* = 47), the median interval was 15.5 months (interquartile range, 11.8–20.5). Patients with more recent BCMA therapy (<1 year, *n* = 15) had inferior OS compared with those treated ≥1 year prior (*n* = 32), with median OS of 4.1 months versus not reached (HR 3.66, 95% CI 1.26–10.6, *p* = 0.017).

Baseline hemoglobin, LDH, and ferritin were consistently associated with treatment outcomes.

Higher baseline hemoglobin was associated with higher odds of response (OR 1.46, 95% CI 1.20–1.81, *p* < 0.001), including both ≥VGPR (OR 1.46, 95% CI 1.20–1.81, *p* < 0.001) and ≥CR (OR 1.54, 95% CI 1.26–1.92, *p* < 0.001). Higher LDH (per 100 U/L; OR 0.78, 95% CI 0.60–0.95, *p* = 0.028) and higher ferritin (per 1000 ng/mL; OR 0.63, 95% CI 0.43–0.88, *p* = 0.013) were associated with lower response rates.

For survival outcomes, higher hemoglobin was associated with superior OS (HR 0.71, 95% CI 0.60–0.83, *p* < 0.001), PFS (HR 0.74, 95% CI 0.64–0.85, *p* < 0.001), DOR (HR 0.77, 95% CI 0.60–0.98, *p* = 0.031), and IFS (HR 0.82, 95% CI 0.73–0.93, *p* < 0.001). Higher LDH and ferritin were each associated with inferior OS (LDH HR 1.37, 95% CI 1.23–1.53, *p* < 0.001; ferritin HR 1.41, 95% CI 1.22–1.62, *p* < 0.001), PFS (LDH HR 1.31, 95% CI 1.18–1.44, *p* < 0.001; ferritin HR 1.34, 95% CI 1.17–1.53, *p* < 0.001), DOR (LDH HR 1.43, 95% CI 1.06–1.93, *p* = 0.020; ferritin HR 1.57, 95% CI 1.17–2.10, *p* = 0.003), and IFS (LDH HR 1.21, 95% CI 1.11–1.31, *p* < 0.001; ferritin HR 1.28, 95% CI 1.11–1.47, *p* < 0.001).

**Fig. 4:**
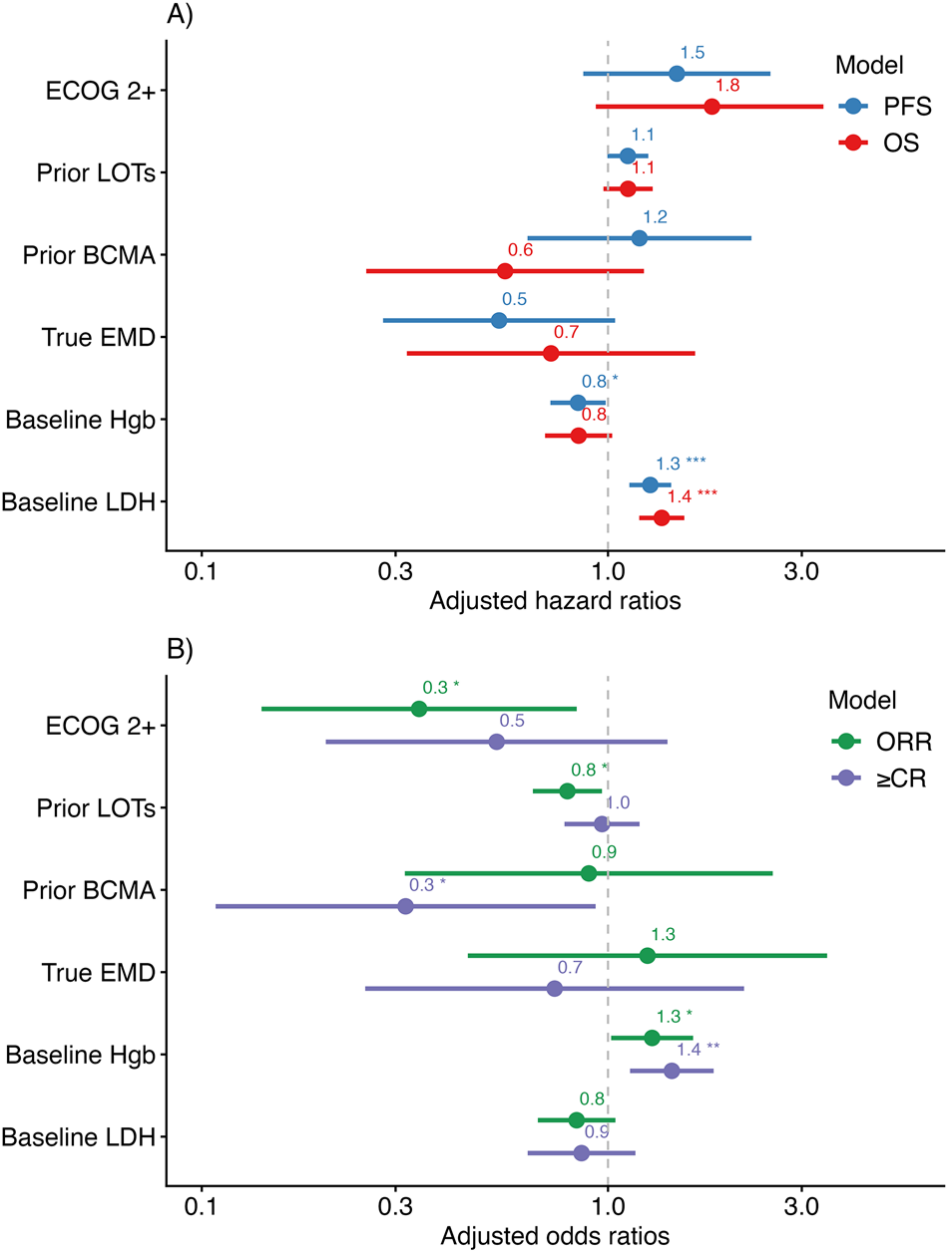
Multivariable predictors of efficacy outcomes. Forest plot depicting multivariable models for **A** progression-free survival (PFS) and overall survival (OS), and **B** achieving an overall response (ORR) and at least a complete response (≥CR). *p < 0.05, **p < 0.01, ***p < 0.001.

In MVA, several associations remained independently significant. Elevated LDH was associated with inferior OS (aHR 1.36, 95% CI 1.19–1.54, *p* < 0.001), PFS (aHR 1.27, 95% CI 1.13–1.43, *p* < 0.001), and IFS (aHR 1.15, 95% CI 1.04–1.28, *p* = 0.006). Higher hemoglobin was associated with higher ORR (aOR 1.28, 95% CI 1.03–1.64, *p* = 0.035) and ≥CR (aOR 1.44, 95% CI 1.14–1.85, *p* = 0.003), as well as superior PFS (aHR 0.84, 95% CI 0.72–0.99, *p* = 0.034) and IFS (aHR 0.84, 95% CI 0.73–0.97, *p* = 0.019). Prior BCMA exposure was associated with lower odds of achieving ≥CR (aOR 0.32, 95% CI 0.10–0.91, *p* = 0.037). Ferritin and BMPC burden (≥50% vs. <50%) were excluded from MVA due to missingness (ferritin, *n* = 19; BMPC burden, *n* = 45). Forest plots of these models are shown in Fig. 4.

To further explore the relationship with outcomes, restricted cubic spline analyses were performed (Supplemental Fig. 6). These revealed a sharp rise in the risk of progression and death below hemoglobin ~11 g/dL, a continuously rising risk of death with higher LDH and ferritin, and diminished probability of achieving ≥CR and greater probability of primary PD at lower hemoglobin levels.

### Toxicity and supportive care

**Fig. 5:**
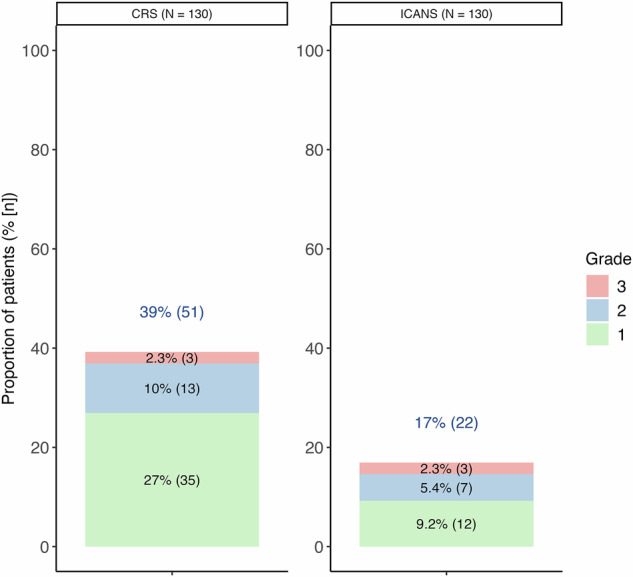
Treatment-emergent CRS and ICANS. Maximum grade cytokine release syndrome (CRS) and immune effector cell-associated neurotoxicity (ICANS).

As shown in Fig. 5, CRS occurred in 40% of patients, with grade ≥2 events in 12.3% and grade 3 in 2.3%. The median time to onset was 2 days (IQR, 1–3; range, 0–15), and the median duration was 1 day (IQR, 1–2; range, 1–4). Recurrent CRS was observed in 14% of patients.

ICANS occurred in 17% of patients, with grade ≥2 events in 7.7%. The median onset was 2 days (IQR, 1–4; range, 0–21), and the median duration was 2 days (IQR, 1–2; range, 1–10).

During the first treatment cycle, ICU admission was required in 6.2% of patients, with a median ICU stay of 4 days (IQR, 2–8). The median inpatient length of stay was 4.5 days, and 3.1% of patients completed treatment entirely as outpatients without hospitalization.

ICANS was less common in patients with prior BCMA exposure (9.4% vs. 24%; OR 0.32, 95% CI 0.11–0.85, *p* = 0.029), more prior LOTs (OR 0.73, 95% CI 0.55–0.92, *p* = 0.016), and higher baseline hemoglobin (OR 0.71, 95% CI 0.52–0.94, *p* = 0.023). Among patients with available baseline BMPC data (*n* = 85), we did not observe a clear association between high marrow burden (≥50% vs. <50%) and the incidence of CRS (any-grade 40% vs. 39%, *p* > 0.9) or ICANS (any-grade 20% vs. 17%, *p* = 0.7).

Tocilizumab was administered to 36% of patients (28% for treatment, 9.2% for prophylaxis), and corticosteroids were used in 23%. Steroid use was less common in BCMA-exposed patients (11% vs. 34%; OR 0.23, 95% CI 0.08–0.60, *p* = 0.004) and those with more prior LOTs (OR 0.68, 95% CI 0.52–0.85, *p* = 0.002).

We observed a numeric trend toward a lower incidence of any-grade CRS among patients who received prophylactic tocilizumab (OR 0.49, 95% CI 0.10–1.73, *p* = 0.30), although this analysis was limited by the small number of patients receiving prophylaxis (*n* = 12). Notably, no cases of ICANS were observed in patients who received prophylactic tocilizumab.

### Impact of IVIg prophylaxis on infections

Infections occurred in 38% of patients (*n* = 49), of whom 57% (*n* = 28) required hospitalization. To assess the impact of IVIg prophylaxis, we modeled its initiation as a time-dependent covariate. IVIg use was associated with significantly longer IFS (HR 0.46, 95% CI 0.25–0.84, *p* = 0.013) and a trend toward improved infection-free PFS (HR 0.58, 95% CI 0.33–1.02, *p* = 0.058). Both associations were significant in MVA: IVIg use was independently associated with prolonged IFS (aHR 0.46, 95% CI 0.24-0.89, *p* = 0.021) and infection-free PFS (aHR 0.54, 95% CI 0.29–0.99, *p* = 0.046).

**Fig. 6:**
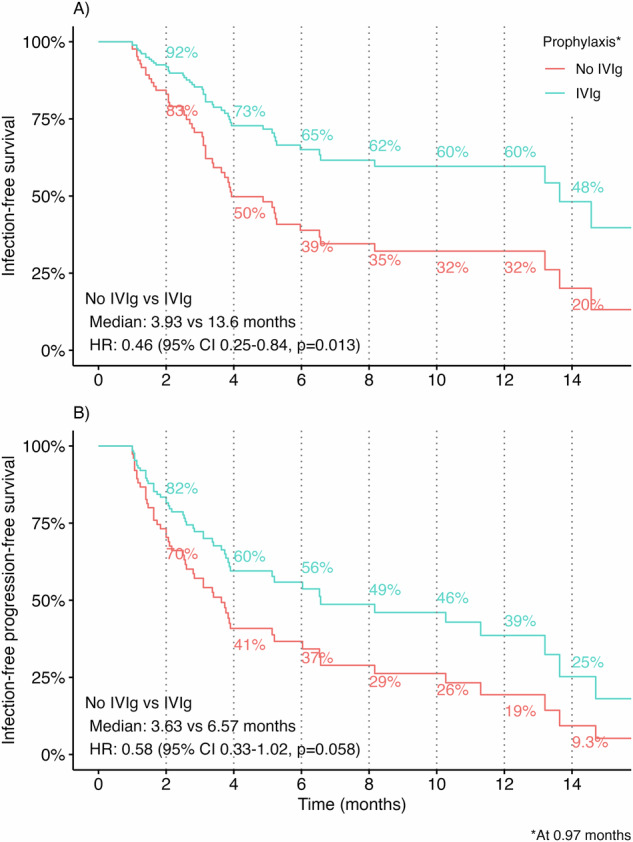
IVIg prophylaxis and infection-related outcomes. Smith-Zee plots for **A** infection-free survival and **B** infection-free progression-free with and without IVIg prophylaxis, modeled as a time-dependent covariate based on the start of IVIg therapy. Curves represent predicted survival probabilities from time-dependent Cox models, with hypothetical patients entering maintenance at 0.97 months after initiating elranatamab (median initiation time of IVIg).

As shown in Fig. 6, Smith-Zee plots were used to visualize predicted survival probabilities, treating IVIG as a time-dependent covariate. The plots illustrate predicted outcomes for hypothetical patients initiating IVIg at the median time of 0.97 months after elranatamab initiation, highlighting early and sustained divergence in infection-free outcomes following IVIg initiation.

### CAR-HEMATOTOX score

The distribution of calculated CAR-HEMATOTOX scores is shown in Supplementary Fig. 7. The median score was 2 (IQR, 1–3; unknown in 28 patients). When analyzed as a continuous variable (per 1-point increase), higher CAR-HEMATOTOX scores were associated with lower rates of ≥VGPR (OR 0.64, 95% CI 0.43–0.92, *p* = 0.020) and ≥CR (OR 0.60, 95% CI 0.40–0.89, *p* = 0.014). Increasing scores were also linked to inferior survival outcomes, including OS (HR 1.64, 95% CI 1.23–2.14, *p* < 0.001), PFS (HR 1.36, 95% CI 1.09–1.70, *p* = 0.007), DOR (HR 1.68, 95% CI 1.16–2.42, *p* = 0.006), and IFS (HR 1.35, 95% CI 1.07–1.72, *p* = 0.013).

### Novel ALPS score

**Fig. 7:**
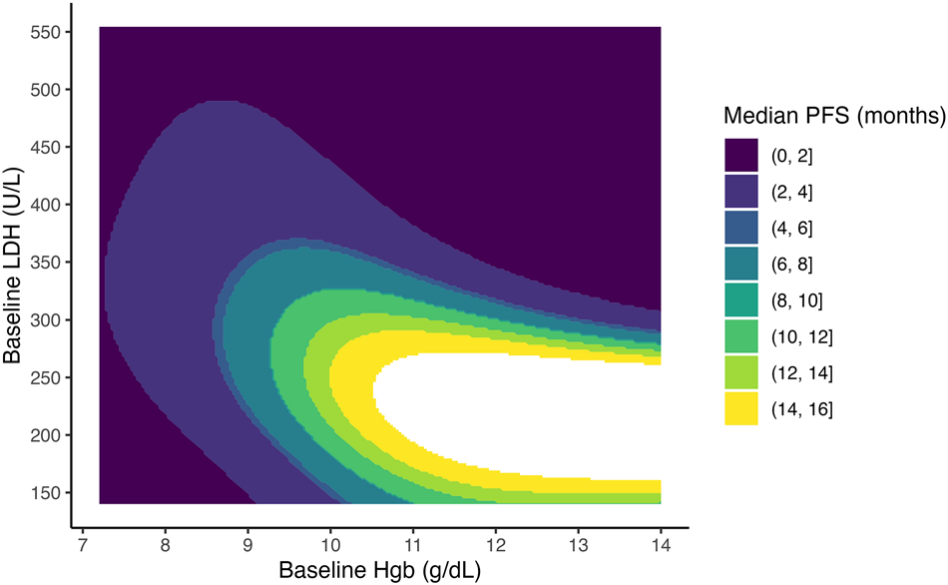
Contour plot illustrating the joint association of baseline hemoglobin (Hgb) and baseline lactate dehydrogenase (LDH) with median progression-free survival (PFS). Restricted cubic spline models with interaction terms were applied to capture potential non-linear effects. Median PFS (in months) is shown by contour shading, with darker regions representing shorter PFS and lighter regions representing longer PFS.

Supplementary Fig. 6 depicts the association of baseline hemoglobin with response (≥CR and PD; panel A), as well as with PFS and OS (panel B), and the association of baseline LDH with PFS and OS (panel C). Consistent with these findings, Fig. 7 shows that lower hemoglobin and higher LDH were jointly associated with inferior PFS, with the poorest outcomes observed in patients with hemoglobin <10 g/dL and LDH > 300 U/L.

Based on these observations, we developed the ALPS (Anemia-LDH Prognostic System) score, assigning one point each for hemoglobin <10 g/dL and LDH > 300 U/L, for a total score ranging from 0 to 2. As illustrated in Supplemental Fig. 8, patients with an ALPS score of 0 had markedly superior outcomes compared to those with a score of 1–2, including OS (median not reached vs. 3.93 months; HR 5.04, 95% CI 2.42-10.48, *p* < 0.001), PFS (median 13.2 vs. 1.73 month; HR 3.10, 95% CI 1.84-5.23, *p* < 0.001), and DOR (median not reached vs. 6.13 months; HR 3.23, 95% CI 1.36–7.64, p = 0.0076). Comprehensive univariate and multivariable regression results incorporating the ALPS score are provided in Supplemental Table 2.

In multivariable analysis (Supplementary Table 3) adjusted for both ALPS score and CAR-HEMATOTOX HT^high^ (≥2 points; reference: HT^low^, 0–1 points), the ALPS score remained independently associated with response (1 point: aOR 0.30, *p* = 0.011; 2 points: aOR 0.10, *p* = 0.001), ≥VGPR (1 point: aOR 0.21, *p* < 0.001; 2 points: aOR 0.08, *p* = 0.003), ≥CR (1 point: aOR 0.26, *p* = 0.004; 2 points: 0.07, *p* = 0.016). Similarly, higher ALPS scores were independently associated with inferior OS (1 point: aHR 6.56, *p* < 0.001; 2 points: aHR 9.42, *p* < 0.001) and PFS (1 point: 3.80, *p* < 0.001; 2 points: aHR 4.83, *p* < 0.001). In contrast, CAR-HEMATOTOX HT^high^ was not independently associated with any clinical outcomes.

To evaluate model discrimination and calibration, we generated time-specific calibration plots for both the ALPS and CAR-HEMATOTOX scores (Supplemental Fig. 9). For PFS, the ALPS score demonstrated superior discrimination with C-indices of 0.69, 0.66, and 0.66 at 1, 3, and 6 months, respectively, compared to 0.60, 0.59, and 0.61 for the CAR-HEMATOTOX score. Corresponding AUCs for ALPS were 0.70, 0.68, and 0.67, versus 0.60, 0.61, and 0.63 for CAR-HEMATOTOX. Similar patterns were observed for OS, with ALPS achieving C-indices of 0.80, 0.70, and 0.73 at 1, 3, and 6 months, respectively, compared to 0.63, 0.66, and 0.69 for CAR-HEMATOTOX. AUC values for ALPS at these timepoints were 0.82, 0.71, and 0.75, versus 0.64, 0.67, and 0.74 for CAR-HEMATOTOX. These findings highlight the superior discriminatory performance and similar calibration of the ALPS score across clinically relevant timepoints.

## Discussion

In this multicenter real-world analysis, we report outcomes for 130 patients treated with commercial elranatamab, highlighting both the clinical activity and the limitations of BCMA-directed BsAb therapy in a frail, heavily pretreated population. Compared to the Phase 2 MagnetisMM-3 trial, which established the efficacy of elranatamab in BCMA-naïve patients, our cohort reflects broader real-world use, including patients with prior BCMA exposure, poor performance status, and substantial comorbidity. Only 22% of our patients would have met the eligibility criteria for MagnetisMM-3 cohort A. That trial excluded patients with prior BCMA therapy and enrolled a relatively fit population, with few patients having ECOG PS 2 (5.7%) and none with PS ≥ 3, and only 15.4% with R-ISS stage III disease [1]. In contrast, 35% of our cohort had ECOG PS ≥ 2, including 24% with PS 2 and 11% with PS 3; 49% had prior BCMA exposure, and 49% were penta-refractory.

Despite these baseline differences, the ORR in our cohort was comparable to that observed in MagnetisMM-3 (65% vs. 61%), as was the ≥CR rate (36% vs. 35%) [1]. However, treatment durability was markedly inferior in the real-world setting: median PFS and OS were 4.3 and 14.6 months, respectively, compared to 17.2 and 24.6 months in MagnetisMM-3 cohort A [2, 3]. These discrepancies likely reflect adverse disease biology, a higher burden of disease, prior BCMA exposure, greater frailty, and reduced tolerance for prolonged therapy in routine clinical practice. Our outcomes also align with a French compassionate-use cohort (n = 101), which reported an ORR of 51.5%, and one-year PFS and OS rates of 34% and 42%, respectively [14]. IVIg utilization and infection rates were similar across cohorts; however, the French study did not evaluate the relationship between IVIg and IFS.

Multivariable modeling identified elevated baseline LDH as a consistent and independent predictor of inferior PFS and OS, with each 100 U/L increase conferring a 27% and 36% increased hazard, respectively. Additionally, lower hemoglobin levels were independently associated with worse response and shorter PFS, with each 1 g/dL decrease conferring a 19% increase in hazard, suggesting that baseline marrow reserve may influence efficacy. These two variables—LDH and hemoglobin—emerged as dominant correlates of poor outcome across multiple endpoints.

Importantly, direct measures of tumor burden further supported this observation. Among patients with available baseline BMPC data, a high marrow burden (≥50%) was associated with markedly inferior response rates and survival, including lower ORR and ≥VGPR rates and substantially shorter PFS and OS. Notably, high BMPC burden was not associated with higher rates of CRS or ICANS, suggesting that disease burden may differentially impact efficacy rather than immune-mediated toxicity. Together, these findings support the hypothesis that elranatamab may be more effective in lower tumor burden disease states, and they raise the possibility that cytoreductive strategies prior to initiation could improve depth and durability of response.

Hemoglobin and LDH emerged as the more robust predictors of outcomes than the CAR-HEMATOTOX score. Leveraging the simplicity and widespread availability of these routinely collected labs, we developed the ALPS (Anemia-LDH Prognostic System) score, assigning 1 point each for hemoglobin <10 g/dL and LDH > 300 U/L (range, 0–2). Patients with an ALPS score of 0 had significantly improved outcomes, including OS (median not reached vs. 3.93 months), PFS (13.2 vs. 1.73 months; HR 3.10, *p* < 0.001), and DOR (not reached vs. 6.13 months; HR 3.23, p = 0.0076). In multivariable models incorporating both ALPS and CAR-HEMATOTOX scores, ALPS remained independently associated with ≥CR, PFS, and OS, whereas the CAR-HEMATOTOX score was not. Further supporting its utility, the ALPS score demonstrated comparable calibration with superior discrimination compared to CAR-HEMATOTOX. Taken together, the ALPS score offers a simple, reproducible, and clinically accessible tool for real-world risk stratification in patients receiving elranatamab.

Prior BCMA exposure emerged as a key determinant of response depth. BCMA-exposed patients had significantly lower odds of achieving ≥CR (aOR 0.32, *p* = 0.037) or ≥VGPR (aOR 0.35, *p* = 0.047), even after adjusting for other factors. While small numbers limited definitive comparisons, patients with more recent BCMA exposure (<1 year) appeared to have worse OS (HR 3.66, *p* = 0.017), consistent with emerging data suggesting a time-dependent impact of prior BCMA treatment. These findings reinforce the need to tailor treatment sequencing based on receipt and timing of prior BCMA-targeted therapies.

Infections were a common complication, affecting 38% of patients and contributing to treatment delays or discontinuation. Overall, 46% of patients received IVIg, and its use (modeled as a time-dependent covariate) was associated with significantly improved IFS (aHR 0.46, *p* = 0.021) and infection-free PFS (aHR 0.54, *p* = 0.046). These findings align with recent studies demonstrating reduced serious infection rates and improved survival with IVIg in patients receiving BCMA-targeted BsAbs [4, 5]. Our analysis extends this evidence by using time-dependent modeling to minimize immortal time bias and to provide more precise estimates of benefit.

Rates of cytokine release syndrome (CRS; 40%, ≥grade 3 in 2.3%) and ICANS (17%) differed from those reported in MagnetisMM-3 (CRS 57.7% any grade, 0% ≥grade 3; ICANS 3.3%) [1]. While the overall incidence of CRS was modestly lower, the rate of ICANS was higher in our real-world cohort. This divergence may reflect a combination of reporting differences and true variation in patient-level risk factors, including greater frailty, comorbidity burden, and disease heterogeneity, as well as differences in supportive care practices. In particular, prophylactic tocilizumab use may have attenuated CRS, as has been reported in real-world studies of bispecific antibodies and other T-cell-redirecting therapies, while its impact on ICANS appears less consistent across cohorts [15–19]. These findings highlight the importance of supportive care measures: 36% of patients required tocilizumab (28% for treatment and 9.2% prophylactically), 23% required corticosteroids, and 24% required G-CSF.

Our study is limited by its retrospective design and inherent variability in institutional practices regarding infection prophylaxis, IVIg administration, and supportive care. In addition, detailed data regarding elranatamab dosing schedules, including dose interruptions, delays, and transitions to less frequent maintenance dosing, were not systematically captured across participating centers, limiting more granular analyses of dose intensity and treatment exposure. Data on IgG levels, cytogenetic risk, and depth of prior responses were incomplete for a subset of patients. Nevertheless, this multicenter cohort represents one of the largest real-world experiences with elranatamab and offers critical insights into efficacy, safety, and supportive care in routine practice.

In summary, elranatamab demonstrates the ability to induce objective responses in a frail and heavily pretreated real-world population; however, the markedly shorter PFS observed in this cohort (4.3 vs. 17.2 months) compared with MagnetisMM-3 cohort A reflects substantial limitations in treatment durability outside of clinical trial settings. These data indicate that while responses can be achieved, sustained disease control is frequently limited in routine practice, particularly among patients with high baseline tumor burden, impaired marrow reserve, and adverse clinical features.

Several considerations emerge from these findings that have direct implications for patient selection. Baseline disease burden appears to be a critical determinant of efficacy, with high marrow plasma cell involvement, anemia, and elevated LDH consistently associated with inferior outcomes. Together, these features identify a subgroup of patients in whom elranatamab appears to have limited effectiveness, suggesting that patients with aggressive, high-volume disease or poor marrow reserve may be less likely to derive durable benefit. Conversely, these data suggest that elranatamab may be better suited for patients with lower tumor burden and preserved marrow function. This raises the possibility that earlier use, cytoreductive approaches prior to initiation, or rational combination strategies may be required to improve depth and durability of response, although these strategies remain unproven. In addition, infectious complications were common, reinforcing the importance of proactive supportive care, particularly IVIg supplementation, which was associated with improved infection-related outcomes.

Finally, although prior BCMA exposure, particularly when recent, was associated with reduced depth and durability of response, these data do not define an optimal sequencing strategy. Rather, they indicate a need for prospective studies to better inform patient selection, timing, and sequencing of elranatamab within the BCMA-directed treatment landscape. Taken together, our findings do not support broad, unselected use of elranatamab in relapsed/refractory multiple myeloma, but instead favor careful patient selection, avoidance in patients with aggressive high-burden disease or limited marrow reserve, and further investigation to identify settings in which durable benefit may be achieved.

## Supplementary information

Supplemental Materials

## Data Availability

The datasets generated during and/or analyzed during the current study are available from the corresponding author on reasonable request.
