# Supplementary material for: Real-world outcomes with elranatamab in multiple myeloma: a multicenter analysis from the U.S. Multiple Myeloma Immunotherapy Consortium
Source: Blood Cancer J. 2026 Mar 27;16(1):47. doi: 10.1038/s41408-026-01477-z (PMC13039266; doi:10.1038/s41408-026-01477-z)
Supplement: Supplementary file 1 — Supplemental Materials [file 41408_2026_1477_MOESM1_ESM.docx]

**TABLES**

**Supplemental Table 1.** Regression output from univariate and multivariable models.

| **Characteristic** | **Univariate** | | | | **Multivariable** | | |
| --- | --- | --- | --- | --- | --- | --- | --- |
|  | **N** | **Estimate***^1^* | **95% CI** | **p-value** | **Estimate** | **95% CI** | **p-value** |
| **Overall Response** | | | | | | | |
| Baseline Hgb | 130 | 1.46 | 1.20, 1.81 | <0.001 | 1.28 | 1.03, 1.64 | 0.035 |
| Baseline LDH | 128 | 0.78 | 0.60, 0.95 | 0.028 | 0.84 | 0.65, 1.02 | 0.11 |
| ECOG 2+ | 126 | 0.34 | 0.16, 0.73 | 0.006 | 0.34 | 0.14, 0.83 | 0.019 |
| True EMD | 129 | 0.89 | 0.38, 2.16 | 0.8 | 1.25 | 0.46, 3.59 | 0.7 |
| Prior LOTs | 129 | 0.84 | 0.73, 0.97 | 0.017 | 0.79 | 0.65, 0.96 | 0.021 |
| Prior BCMA | 130 | 0.60 | 0.29, 1.22 | 0.2 | 0.90 | 0.32, 2.58 | 0.8 |
| **≥VGPR** | | | | | | | |
| Baseline Hgb | 130 | 1.50 | 1.24, 1.86 | <0.001 | 1.40 | 1.12, 1.79 | 0.005 |
| Baseline LDH | 128 | 0.66 | 0.44, 0.89 | 0.022 | 0.80 | 0.55, 1.02 | 0.13 |
| ECOG 2+ | 126 | 0.54 | 0.25, 1.15 | 0.11 | 0.67 | 0.26, 1.70 | 0.4 |
| True EMD | 129 | 0.33 | 0.12, 0.80 | 0.020 | 0.37 | 0.12, 1.04 | 0.068 |
| Prior LOTs | 129 | 0.87 | 0.75, 1.0 | 0.049 | 0.89 | 0.72, 1.09 | 0.3 |
| Prior BCMA | 130 | 0.38 | 0.19, 0.77 | 0.008 | 0.35 | 0.12, 0.97 | 0.047 |
| **CR/sCR** | | | | | | | |
| Baseline Hgb | 130 | 1.54 | 1.26, 1.92 | <0.001 | 1.44 | 1.14, 1.85 | 0.003 |
| Baseline LDH | 128 | 0.70 | 0.45, 0.94 | 0.057 | 0.86 | 0.59, 1.11 | 0.3 |
| ECOG 2+ | 126 | 0.46 | 0.19, 1.03 | 0.067 | 0.53 | 0.20, 1.38 | 0.2 |
| True EMD | 129 | 0.55 | 0.20, 1.37 | 0.2 | 0.74 | 0.24, 2.11 | 0.6 |
| Prior LOTs | 129 | 0.92 | 0.79, 1.06 | 0.2 | 0.97 | 0.77, 1.19 | 0.8 |
| Prior BCMA | 130 | 0.43 | 0.20, 0.89 | 0.025 | 0.32 | 0.10, 0.91 | 0.037 |
| **Overall Survival** | | | | | | | |
| Baseline Hgb | 123 | 0.71 | 0.60, 0.83 | <0.001 | 0.85 | 0.70, 1.02 | 0.086 |
| Baseline LDH | 121 | 1.37 | 1.23, 1.53 | <0.001 | 1.36 | 1.19, 1.54 | <0.001 |
| ECOG 2+ | 119 | 2.41 | 1.33, 4.35 | 0.004 | 1.80 | 0.93, 3.49 | 0.079 |
| True EMD | 122 | 1.07 | 0.52, 2.24 | 0.8 | 0.72 | 0.32, 1.64 | 0.4 |
| Prior LOTs | 122 | 0.99 | 0.89, 1.11 | 0.9 | 1.12 | 0.97, 1.29 | 0.11 |
| Prior BCMA | 123 | 0.67 | 0.37, 1.21 | 0.2 | 0.56 | 0.25, 1.23 | 0.15 |
| **Progression-free Survival** | | | | | | | |
| Baseline Hgb | 124 | 0.74 | 0.64, 0.85 | <0.001 | 0.84 | 0.72, 0.99 | 0.034 |
| Baseline LDH | 122 | 1.31 | 1.18, 1.44 | <0.001 | 1.27 | 1.13, 1.43 | <0.001 |
| ECOG 2+ | 120 | 1.77 | 1.09, 2.87 | 0.020 | 1.48 | 0.87, 2.51 | 0.15 |
| True EMD | 123 | 0.84 | 0.46, 1.54 | 0.6 | 0.54 | 0.28, 1.04 | 0.066 |
| Prior LOTs | 123 | 1.05 | 0.96, 1.14 | 0.3 | 1.12 | 1.00, 1.26 | 0.057 |
| Prior BCMA | 124 | 1.23 | 0.77, 1.98 | 0.4 | 1.20 | 0.63, 2.25 | 0.6 |
| **Duration of Response** | | | | | | | |
| Baseline Hgb | 66 | 0.77 | 0.60, 0.98 | 0.031 | 0.87 | 0.66, 1.16 | 0.3 |
| Baseline LDH | 64 | 1.43 | 1.06, 1.93 | 0.020 | 1.39 | 0.92, 2.10 | 0.12 |
| ECOG 2+ | 63 | 1.70 | 0.71, 4.07 | 0.2 | 0.96 | 0.32, 2.82 | >0.9 |
| True EMD | 66 | 1.16 | 0.39, 3.45 | 0.8 | 0.54 | 0.14, 2.05 | 0.4 |
| Prior LOTs | 66 | 0.89 | 0.73, 1.09 | 0.3 | 1.05 | 0.76, 1.46 | 0.8 |
| Prior BCMA | 66 | 0.61 | 0.26, 1.44 | 0.3 | 0.67 | 0.21, 2.13 | 0.5 |
| **Infection-free Survival** | | | | | | | |
| Baseline Hgb | 125 | 0.82 | 0.73, 0.93 | 0.001 | 0.84 | 0.73, 0.97 | 0.019 |
| Baseline LDH | 123 | 1.21 | 1.11, 1.31 | <0.001 | 1.15 | 1.04, 1.28 | 0.006 |
| ECOG 2+ | 121 | 1.37 | 0.87, 2.18 | 0.2 | 0.96 | 0.56, 1.62 | 0.9 |
| True EMD | 124 | 1.20 | 0.69, 2.09 | 0.5 | 1.10 | 0.61, 2.00 | 0.7 |
| Prior LOTs | 124 | 0.94 | 0.86, 1.03 | 0.2 | 0.96 | 0.85, 1.08 | 0.5 |
| Prior BCMA | 125 | 0.70 | 0.45, 1.09 | 0.12 | 0.80 | 0.44, 1.45 | 0.5 |
| **ICANS 1+** | | | | | | | |
| Baseline Hgb | 130 | 0.81 | 0.63, 1.03 | 0.094 | 0.83 | 0.62, 1.09 | 0.2 |
| Baseline LDH | 128 | 1.07 | 0.86, 1.29 | 0.5 | 0.98 | 0.75, 1.21 | 0.9 |
| ECOG 2+ | 126 | 2.15 | 0.84, 5.53 | 0.11 | 1.42 | 0.49, 4.02 | 0.5 |
| True EMD | 129 | 1.91 | 0.66, 5.17 | 0.2 | 1.76 | 0.54, 5.31 | 0.3 |
| Prior LOTs | 129 | 0.74 | 0.57, 0.92 | 0.013 | 0.80 | 0.59, 1.03 | 0.11 |
| Prior BCMA | 130 | 0.32 | 0.11, 0.85 | 0.029 | 0.70 | 0.19, 2.52 | 0.6 |
| **CRS 1+** | | | | | | | |
| Baseline Hgb | 130 | 1.03 | 0.87, 1.23 | 0.7 | 1.05 | 0.86, 1.28 | 0.7 |
| Baseline LDH | 128 | 0.95 | 0.78, 1.13 | 0.6 | 0.95 | 0.76, 1.14 | 0.6 |
| ECOG 2+ | 126 | 1.37 | 0.65, 2.89 | 0.4 | 1.44 | 0.64, 3.25 | 0.4 |
| True EMD | 129 | 0.67 | 0.26, 1.58 | 0.4 | 0.58 | 0.22, 1.45 | 0.3 |
| Prior LOTs | 129 | 0.88 | 0.75, 1.01 | 0.069 | 0.87 | 0.72, 1.05 | 0.2 |
| Prior BCMA | 130 | 0.76 | 0.37, 1.54 | 0.4 | 1.28 | 0.51, 3.27 | 0.6 |
| **Steroid use** | | | | | | | |
| Baseline Hgb | 118 | 1.00 | 0.81, 1.24 | >0.9 | 1.04 | 0.81, 1.34 | 0.7 |
| Baseline LDH | 116 | 1.09 | 0.88, 1.32 | 0.4 | 1.06 | 0.82, 1.32 | 0.6 |
| ECOG 2+ | 115 | 1.20 | 0.49, 2.89 | 0.7 | 0.84 | 0.30, 2.30 | 0.7 |
| True EMD | 118 | 2.18 | 0.81, 5.63 | 0.11 | 2.20 | 0.73, 6.45 | 0.2 |
| Prior LOTs | 118 | 0.68 | 0.52, 0.85 | 0.002 | 0.77 | 0.55, 1.01 | 0.081 |
| Prior BCMA | 118 | 0.23 | 0.08, 0.60 | 0.004 | 0.51 | 0.13, 1.79 | 0.3 |
| **Infection** | | | | | | | |
| Baseline Hgb | 129 | 0.93 | 0.78, 1.11 | 0.4 | 0.91 | 0.74, 1.11 | 0.4 |
| Baseline LDH | 127 | 1.06 | 0.89, 1.26 | 0.5 | 1.02 | 0.84, 1.23 | 0.9 |
| ECOG 2+ | 125 | 0.88 | 0.40, 1.86 | 0.7 | 0.73 | 0.31, 1.65 | 0.5 |
| True EMD | 129 | 0.88 | 0.36, 2.08 | 0.8 | 0.90 | 0.34, 2.23 | 0.8 |
| Prior LOTs | 129 | 0.90 | 0.78, 1.03 | 0.15 | 0.91 | 0.75, 1.09 | 0.3 |
| Prior BCMA | 129 | 0.59 | 0.29, 1.21 | 0.2 | 0.79 | 0.31, 2.01 | 0.6 |
| *^1^* Estimate = Odds ratio or hazard ratio | | | | | | | |

**Supplemental Table 2.** Regression output from univariate and multivariable models incorporating the ALPS (Anemia-LDH Prognostic System) score.

| **Characteristic** | **Univariate** | | | | **Multivariable** | | |
| --- | --- | --- | --- | --- | --- | --- | --- |
|  | **N** | **Estimate***^1^* | **95% CI** | **p-value** | **Estimate** | **95% CI** | **p-value** |
| **Overall Response** | | | | | | | |
| ALPS | 129 |  |  |  |  |  |  |
| 0 points |  | — | — |  | — | — |  |
| 1 point |  | 0.37 | 0.16, 0.80 | 0.014 | 0.52 | 0.22, 1.23 | 0.14 |
| 2 points |  | 0.12 | 0.03, 0.42 | 0.002 | 0.16 | 0.03, 0.64 | 0.012 |
| ECOG 2+ | 126 | 0.34 | 0.16, 0.73 | 0.006 | 0.35 | 0.14, 0.84 | 0.020 |
| True EMD | 129 | 0.89 | 0.38, 2.16 | 0.8 | 1.40 | 0.52, 3.98 | 0.5 |
| Prior LOTs | 129 | 0.84 | 0.73, 0.97 | 0.017 | 0.80 | 0.65, 0.96 | 0.024 |
| Prior BCMA | 130 | 0.60 | 0.29, 1.22 | 0.2 | 0.92 | 0.33, 2.56 | 0.9 |
| **≥VGPR** | | | | | | | |
| ALPS | 129 |  |  |  |  |  |  |
| 0 points |  | — | — |  | — | — |  |
| 1 point |  | 0.30 | 0.14, 0.64 | 0.002 | 0.35 | 0.15, 0.81 | 0.016 |
| 2 points |  | 0.10 | 0.02, 0.42 | 0.005 | 0.11 | 0.02, 0.54 | 0.012 |
| ECOG 2+ | 126 | 0.54 | 0.25, 1.15 | 0.11 | 0.62 | 0.25, 1.51 | 0.3 |
| True EMD | 129 | 0.33 | 0.12, 0.80 | 0.020 | 0.35 | 0.12, 0.96 | 0.050 |
| Prior LOTs | 129 | 0.87 | 0.75, 1.0 | 0.049 | 0.90 | 0.73, 1.10 | 0.3 |
| Prior BCMA | 130 | 0.38 | 0.19, 0.77 | 0.008 | 0.40 | 0.14, 1.06 | 0.067 |
| **CR/sCR** | | | | | | | |
| ALPS | 129 |  |  |  |  |  |  |
| 0 points |  | — | — |  | — | — |  |
| 1 point |  | 0.31 | 0.14, 0.68 | 0.004 | 0.39 | 0.16, 0.90 | 0.030 |
| 2 points |  | 0.08 | 0.00, 0.42 | 0.016 | 0.10 | 0.01, 0.60 | 0.036 |
| ECOG 2+ | 126 | 0.46 | 0.19, 1.03 | 0.067 | 0.50 | 0.19, 1.25 | 0.15 |
| True EMD | 129 | 0.55 | 0.20, 1.37 | 0.2 | 0.70 | 0.23, 1.95 | 0.5 |
| Prior LOTs | 129 | 0.92 | 0.79, 1.06 | 0.2 | 0.96 | 0.77, 1.18 | 0.7 |
| Prior BCMA | 130 | 0.43 | 0.20, 0.89 | 0.025 | 0.37 | 0.13, 1.01 | 0.057 |
| **Overall Survival** | | | | | | | |
| ALPS | 122 |  |  |  |  |  |  |
| 0 points |  | — | — |  | — | — |  |
| 1 point |  | 4.70 | 2.20, 10.0 | <0.001 | 4.27 | 1.91, 9.57 | <0.001 |
| 2 points |  | 6.62 | 2.60, 16.9 | <0.001 | 6.18 | 2.21, 17.3 | <0.001 |
| ECOG 2+ | 119 | 2.41 | 1.33, 4.35 | 0.004 | 1.75 | 0.92, 3.32 | 0.089 |
| True EMD | 122 | 1.07 | 0.52, 2.24 | 0.8 | 0.76 | 0.34, 1.67 | 0.5 |
| Prior LOTs | 122 | 0.99 | 0.89, 1.11 | 0.9 | 1.04 | 0.90, 1.19 | 0.6 |
| Prior BCMA | 123 | 0.67 | 0.37, 1.21 | 0.2 | 0.67 | 0.31, 1.42 | 0.3 |
| **Progression-free Survival** | | | | | | | |
| ALPS | 123 |  |  |  |  |  |  |
| 0 points |  | — | — |  | — | — |  |
| 1 point |  | 2.98 | 1.72, 5.17 | <0.001 | 2.67 | 1.51, 4.74 | <0.001 |
| 2 points |  | 3.70 | 1.78, 7.66 | <0.001 | 3.49 | 1.61, 7.53 | 0.001 |
| ECOG 2+ | 120 | 1.77 | 1.09, 2.87 | 0.020 | 1.71 | 1.03, 2.85 | 0.040 |
| True EMD | 123 | 0.84 | 0.46, 1.54 | 0.6 | 0.60 | 0.32, 1.14 | 0.12 |
| Prior LOTs | 123 | 1.05 | 0.96, 1.14 | 0.3 | 1.05 | 0.93, 1.19 | 0.4 |
| Prior BCMA | 124 | 1.23 | 0.77, 1.98 | 0.4 | 1.26 | 0.68, 2.36 | 0.5 |
| *^1^* Estimate = Odds ratio or hazard ratio | | | | | | | |

**Supplemental Table 3.** Univariate and multivariable regression analyses evaluating the association of ALPS (Anemia-LDH Prognostic System) and CAR-HEMATOTOX HT^high^ (≥2 points; reference: HT^low^, score 0-1 points) with clinical outcomes.

| **Characteristic** | **Univariate** | | | | **Multivariable** | | |
| --- | --- | --- | --- | --- | --- | --- | --- |
|  | **N** | **Estimate***^1^* | **95% CI** | **p-value** | **Estimate** | **95% CI** | **p-value** |
| **Overall Response** | | | | | | | |
| ALPS | 129 |  |  |  |  |  |  |
| 0 points |  | — | — |  | — | — |  |
| 1 point |  | 0.37 | 0.16, 0.80 | 0.014 | 0.30 | 0.12, 0.74 | 0.011 |
| 2 points |  | 0.12 | 0.03, 0.42 | 0.002 | 0.10 | 0.02, 0.39 | 0.001 |
| HT^high^ | 113 | 0.65 | 0.21, 1.78 | 0.4 | 1.11 | 0.34, 3.42 | 0.9 |
| **≥VGPR** | | | | | | | |
| ALPS | 129 |  |  |  |  |  |  |
| 0 points |  | — | — |  | — | — |  |
| 1 point |  | 0.30 | 0.14, 0.64 | 0.002 | 0.21 | 0.09, 0.50 | <0.001 |
| 2 points |  | 0.10 | 0.02, 0.42 | 0.005 | 0.08 | 0.01, 0.36 | 0.003 |
| HT^high^ | 113 | 0.59 | 0.22, 1.56 | 0.3 | 1.04 | 0.35, 3.10 | >0.9 |
| **CR/sCR** | | | | | | | |
| ALPS | 129 |  |  |  |  |  |  |
| 0 points |  | — | — |  | — | — |  |
| 1 point |  | 0.31 | 0.14, 0.68 | 0.004 | 0.26 | 0.10, 0.63 | 0.004 |
| 2 points |  | 0.08 | 0.00, 0.42 | 0.016 | 0.07 | 0.00, 0.43 | 0.016 |
| HT^high^ | 113 | 0.55 | 0.21, 1.51 | 0.2 | 0.89 | 0.31, 2.64 | 0.8 |
| **Overall Survival** | | | | | | | |
| ALPS | 122 |  |  |  |  |  |  |
| 0 points |  | — | — |  | — | — |  |
| 1 point |  | 4.70 | 2.20, 10.0 | <0.001 | 6.56 | 2.47, 17.4 | <0.001 |
| 2 points |  | 6.62 | 2.60, 16.9 | <0.001 | 9.42 | 3.04, 29.2 | <0.001 |
| HT^high^ | 106 | 2.07 | 0.73, 5.83 | 0.2 | 1.21 | 0.42, 3.51 | 0.7 |
| **Progression-free Survival** | | | | | | | |
| ALPS | 123 |  |  |  |  |  |  |
| 0 points |  | — | — |  | — | — |  |
| 1 point |  | 2.98 | 1.72, 5.17 | <0.001 | 3.80 | 1.98, 7.31 | <0.001 |
| 2 points |  | 3.70 | 1.78, 7.66 | <0.001 | 4.83 | 2.12, 11.0 | <0.001 |
| HT^high^ | 107 | 1.37 | 0.65, 2.89 | 0.4 | 0.80 | 0.36, 1.76 | 0.6 |
| **Duration of Response** | | | | | | | |
| ALPS | 65 |  |  |  |  |  |  |
| 0 points |  | — | — |  | — | — |  |
| 1 point |  | 4.45 | 1.77, 11.2 | 0.002 | 4.34 | 1.55, 12.1 | 0.005 |
| 2 points |  | 1.27 | 0.26, 6.15 | 0.8 | 1.37 | 0.26, 7.22 | 0.7 |
| HT^high^ | 56 | 2.03 | 0.47, 8.79 | 0.3 | 1.39 | 0.30, 6.36 | 0.7 |
| **Infection-free Survival** | | | | | | | |
| ALPS | 124 |  |  |  |  |  |  |
| 0 points |  | — | — |  | — | — |  |
| 1 point |  | 2.37 | 1.44, 3.88 | <0.001 | 2.53 | 1.44, 4.44 | 0.001 |
| 2 points |  | 2.33 | 1.14, 4.77 | 0.021 | 2.70 | 1.24, 5.87 | 0.012 |
| HT^high^ | 108 | 1.03 | 0.55, 1.93 | >0.9 | 0.73 | 0.37, 1.41 | 0.3 |
| *^1^* Estimate = Odds ratio or hazard ratio | | | | | | | |

**FIGURES**


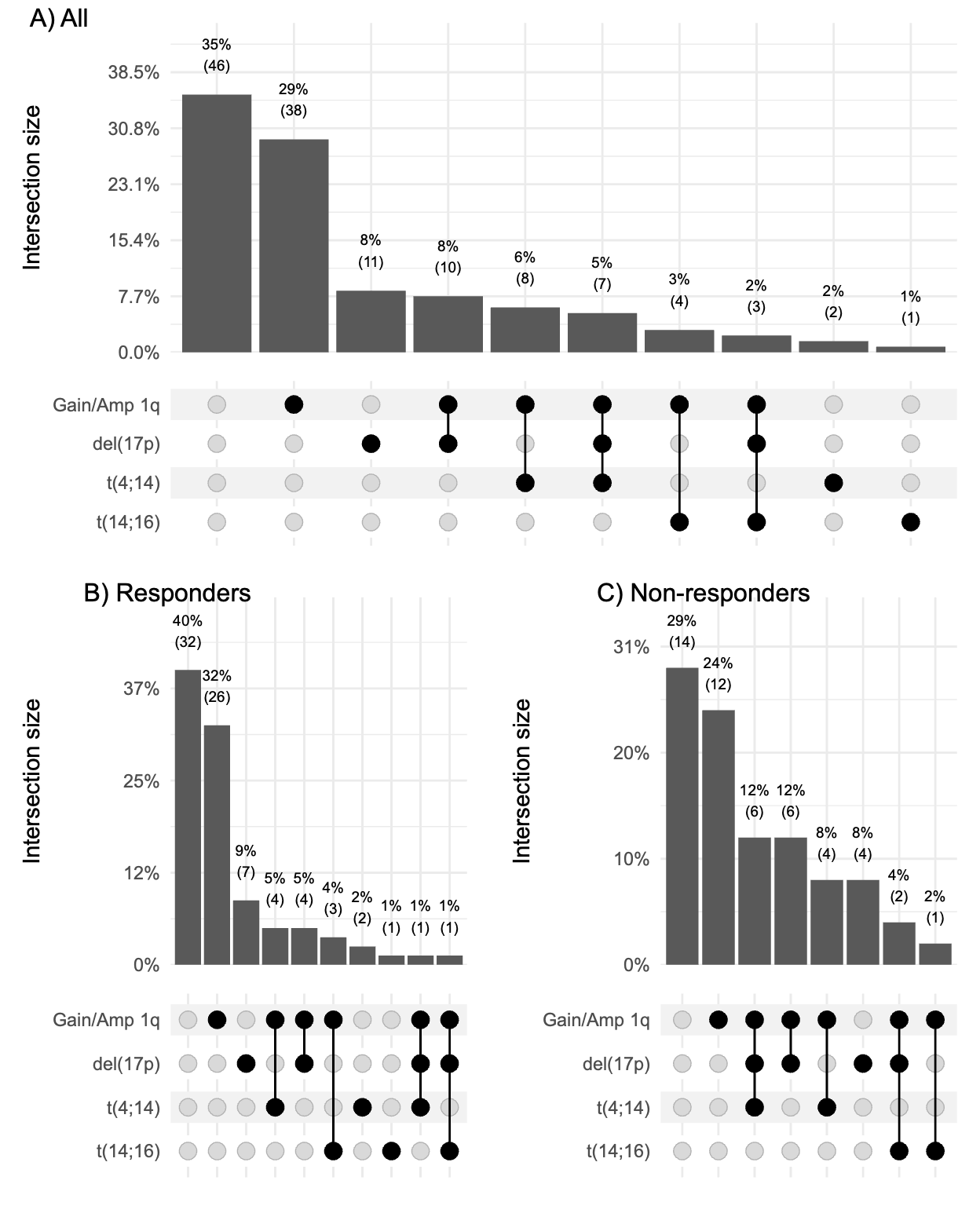


**Supplemental Figure 1.** Upset plot depicting combinations and frequencies of cytogenetic abnormalities in (A) all patients, (B) responders, and (C) non-responders.


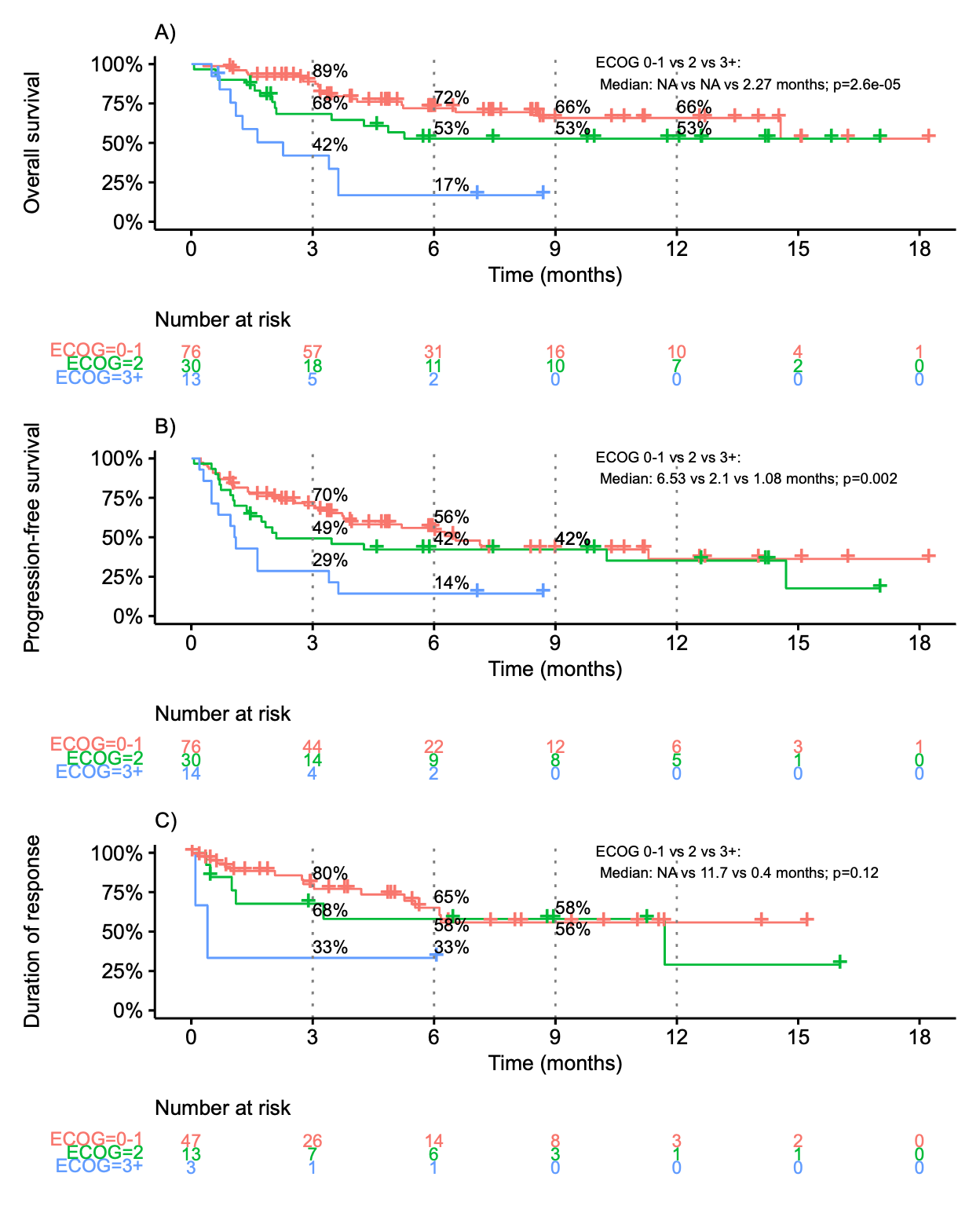


**Supplemental Figure 2.** Kaplan-Meier plots of (A) overall survival, (B) progression-free survival, and (C) duration of response, stratified ECOG performance status.


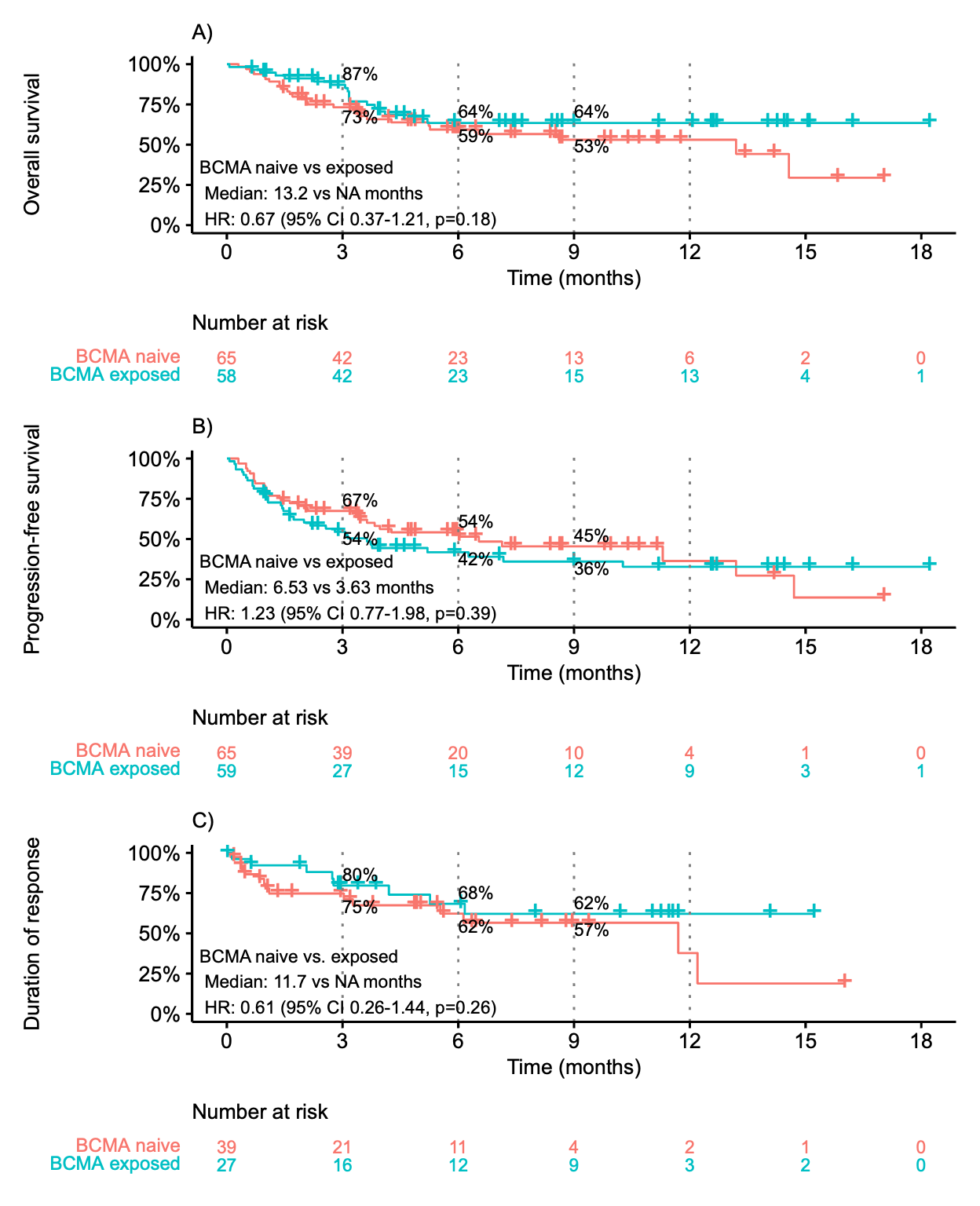


**Supplemental Figure 3.** Kaplan-Meier plots of (A) overall survival, (B) progression-free survival, and (C) duration of response, stratified by prior anti-BCMA therapy exposure.


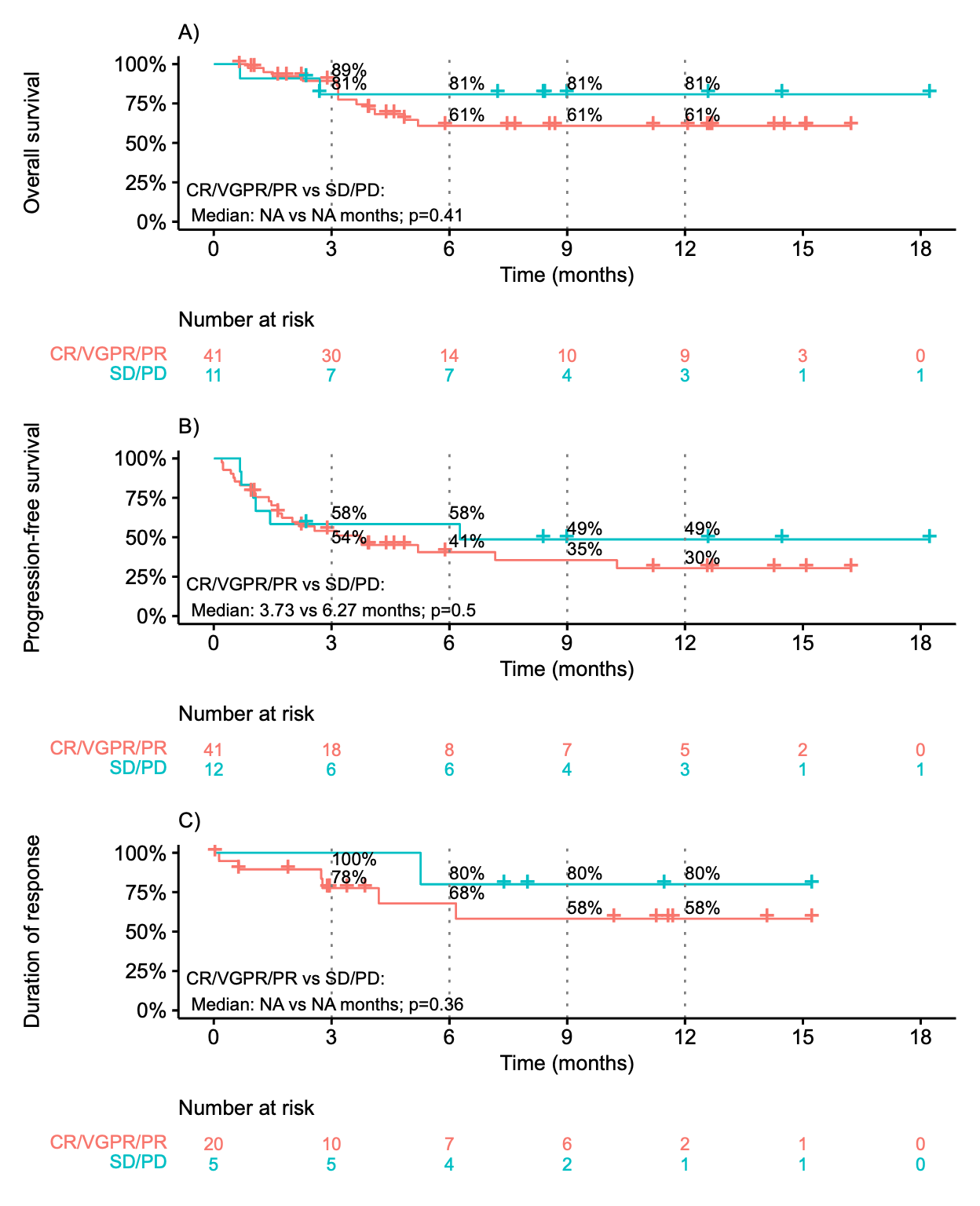


**Supplemental Figure 4.** Kaplan-Meier plots of (A) overall survival, (B) progression-free survival, and (C) duration of response, stratified by response to prior anti-BCMA therapy exposure.


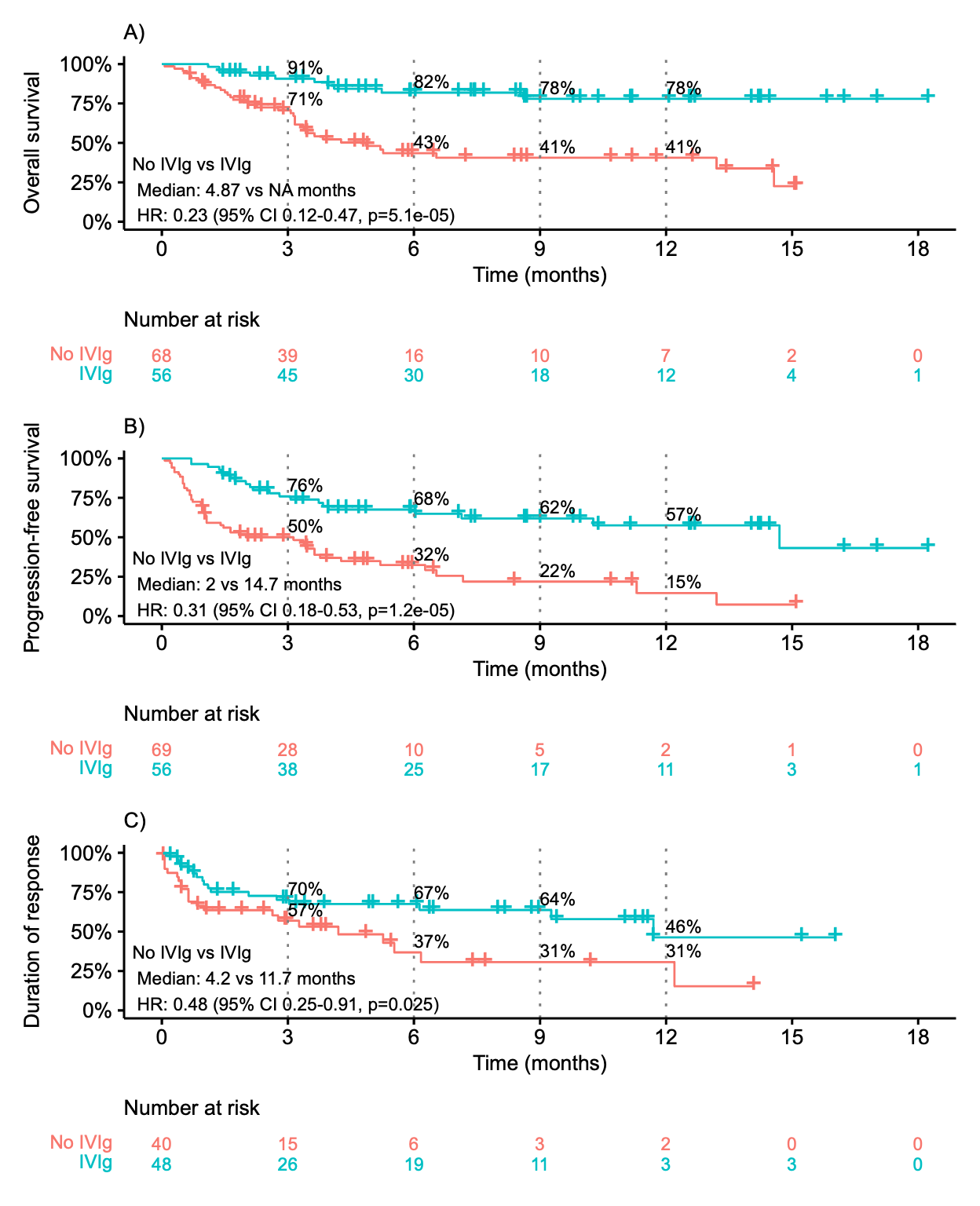


**Supplemental Figure 5.** Kaplan-Meier plots of (A) overall survival, (B) progression-free survival, and (C) duration of response, stratified by receipt of IVIg.


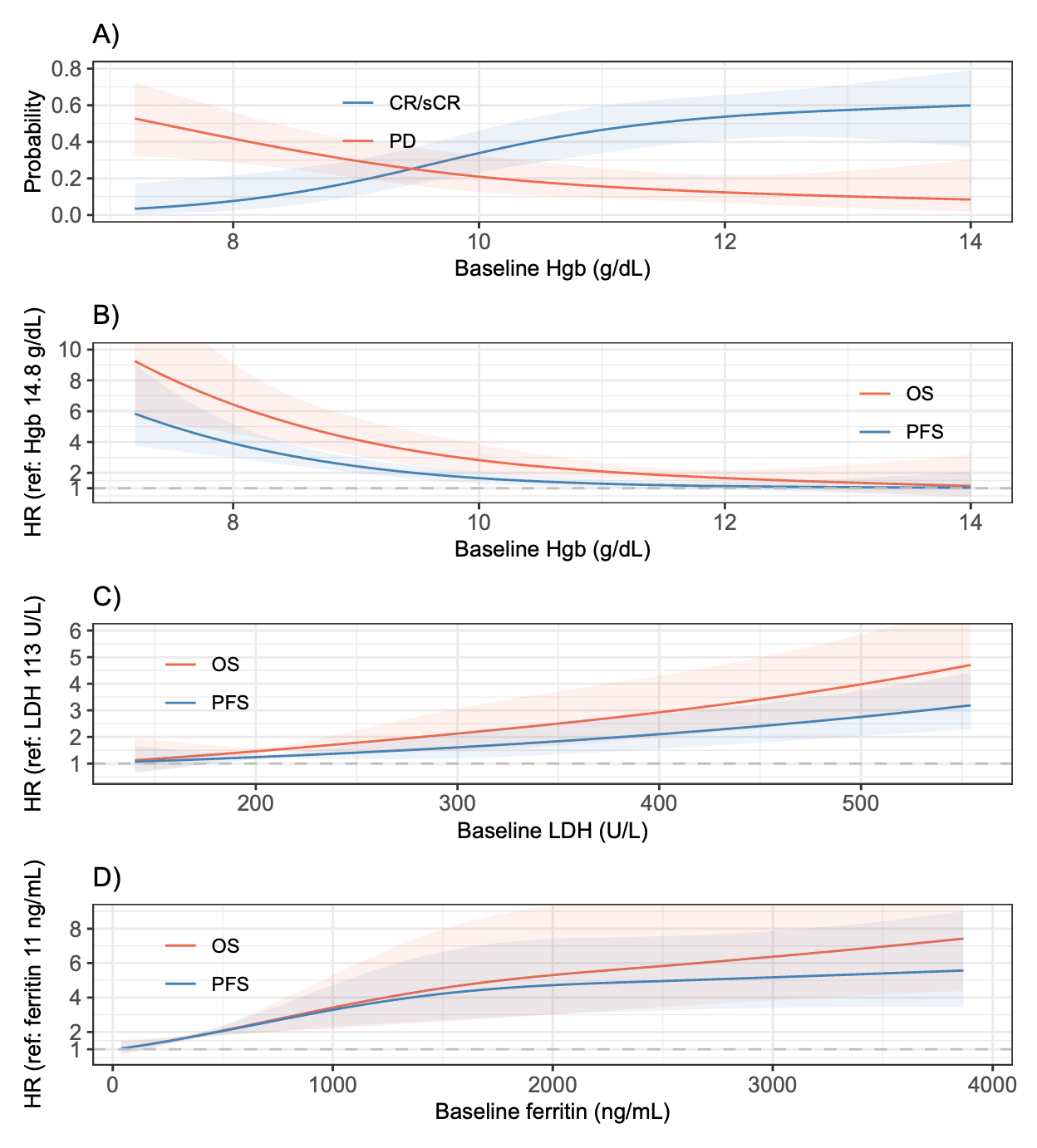


**Supplemental Figure 6.** Restricted cubic spline analyses evaluating baseline biomarkers and clinical outcomes. (A) Probability of CR/sCR or PD according to baseline hemoglobin (Hgb). (B) Association between baseline Hgb and hazard ratios (HRs) for overall survival (OS) and progression-free survival (PFS), referenced to Hgb 14.8 g/dL. (C) Association between baseline LDH and HRs for OS and PFS, referenced to LDH 113 U/L. (D) Association between baseline ferritin and HRs for OS and PFS, referenced to ferritin 11 ng/mL. Reference values were selected to normalize the minimum HRs to 1. Solid lines represent predicted probabilities or HRs, and shaded areas indicate 95% confidence intervals.


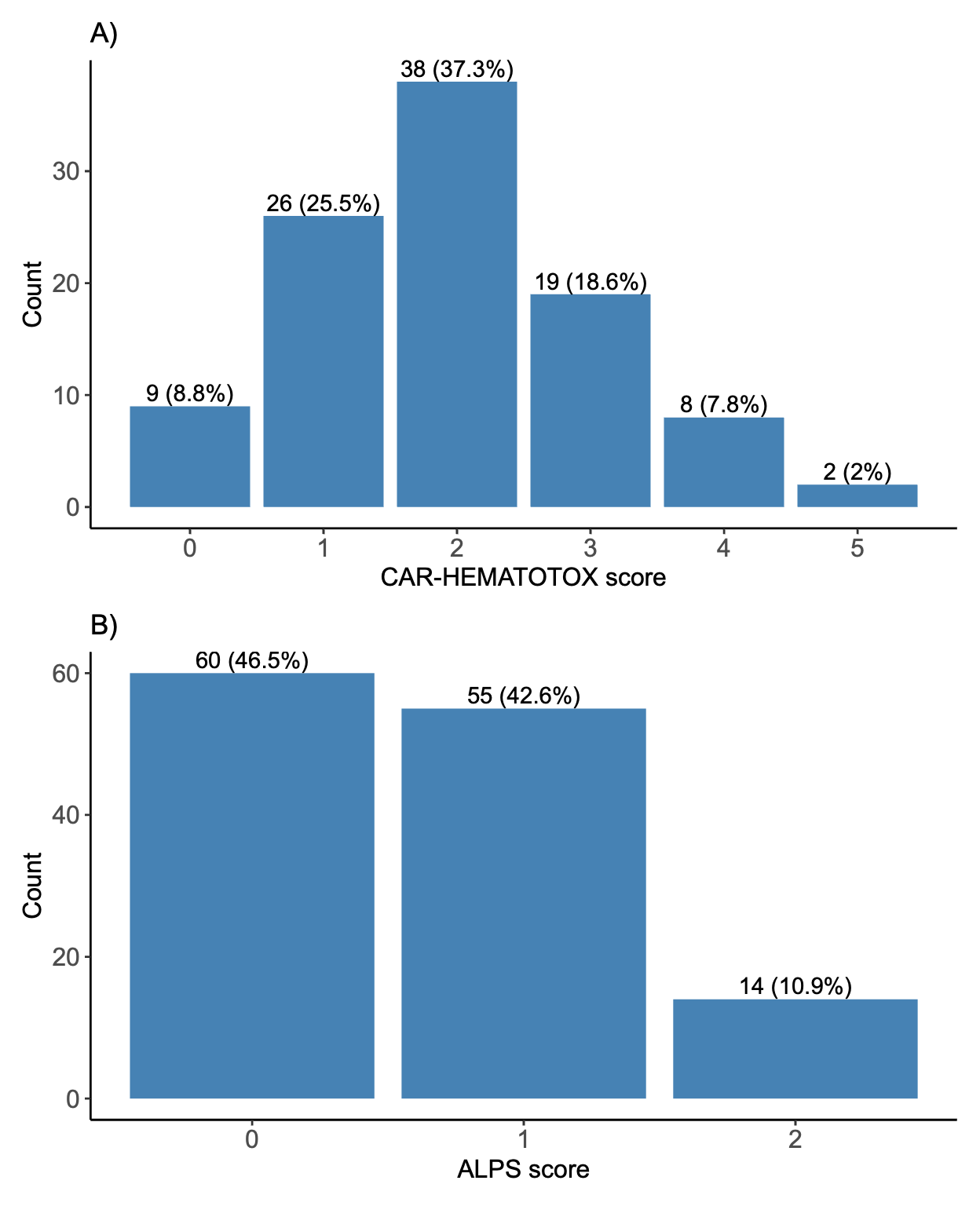


**Supplemental Figure 7.** Distribution of (A) CAR-HEMATOTOX and (B) ALPS (Anemia-LDH Prognostic System) scores.


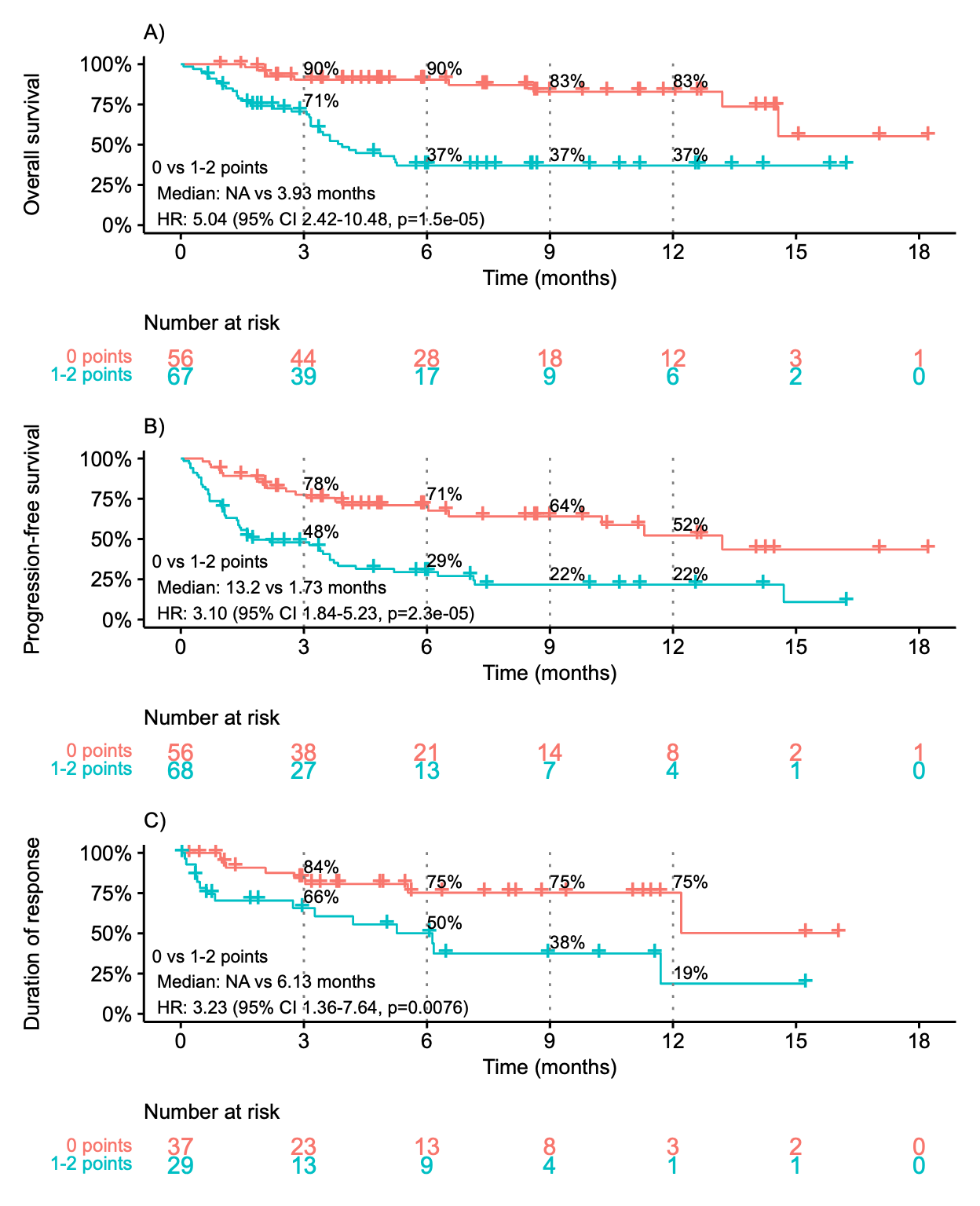


**Supplemental Figure 8.** Kaplan-Meier plots of (A) overall survival, (B) progression-free survival, and (C) duration of response, stratified by ALPS (Anemia-LDH Prognostic System) score of 0 vs. 1-2 points.


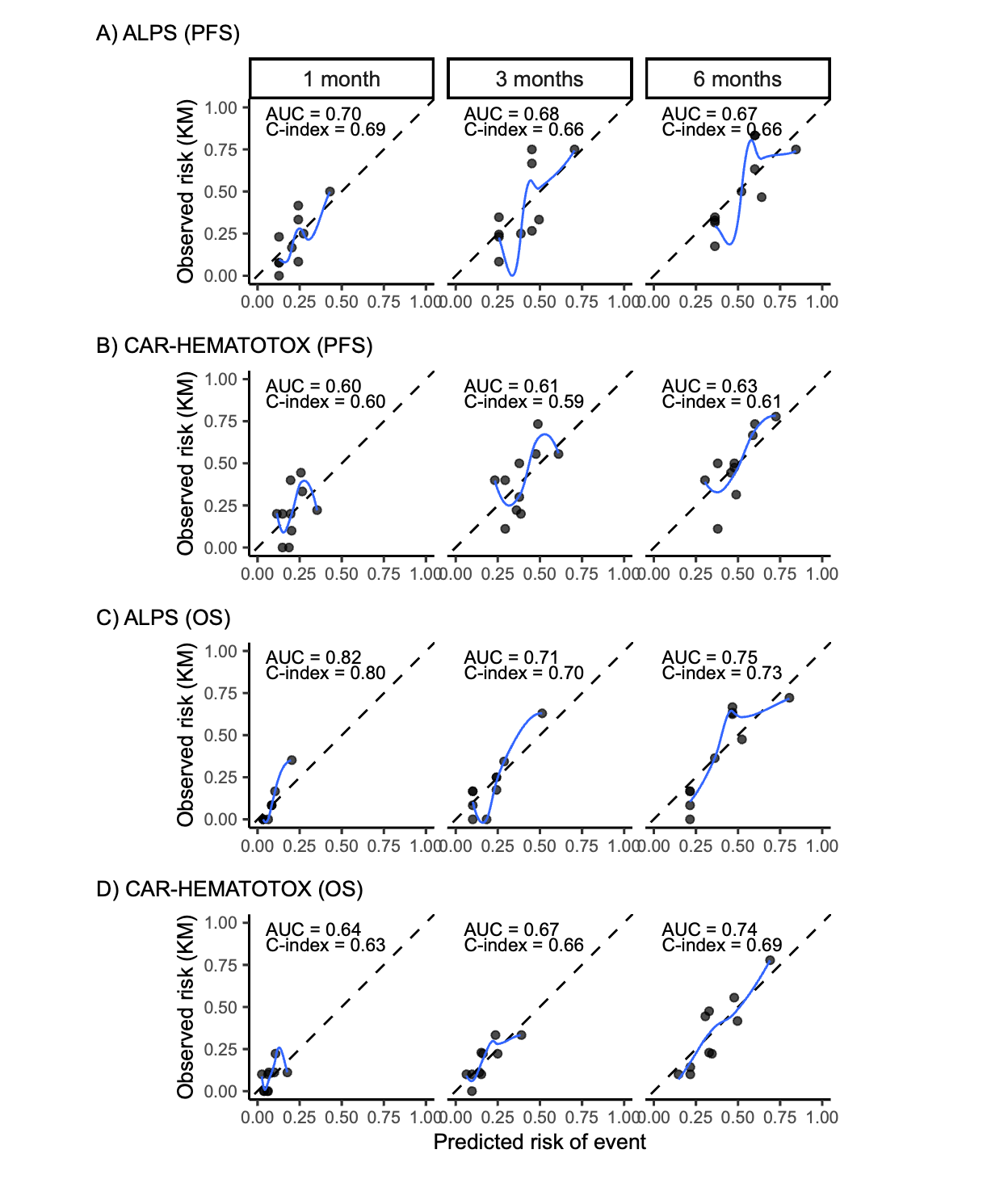


**Supplemental Figure 9.** Calibration plots for ALPS (Anemia-LDH Prognostic System) and CAR-HEMATOTOX scores. Each panel shows the agreement between predicted and observed risk for progression-free survival (PFS) and overall survival (OS), stratified by timepoint. Plots display smoothed LOESS curves against a reference diagonal line representing perfect calibration. Time-specific C-index values and corresponding area under the curve (AUC) estimates are shown in each panel. Plot titles (A-D) denote individual score-outcome combinations.
